# *Burkholderia cenocepacia*-mediated inhibition of *Staphylococcus aureus* growth and biofilm formation

**DOI:** 10.1128/jb.00116-23

**Published:** 2025-03-27

**Authors:** Tiffany J. Brandt, Hayden Skaggs, Thomas Hundley, Deborah R. Yoder-Himes

**Affiliations:** 1 Department of Biology, University of Louisville, Louisville, Kentucky, USA; University of Massachusetts Chan Medical School, Worcester, Massachusetts, USA

**Keywords:** *Burkholderia cenocepacia*, *Staphylococcus aureus*, biofilm, inhibition

## Abstract

*Staphylococcus aureus* asymptomatically colonizes the nasal cavity and pharynx of up to 60% of the human population and, as an opportunistic pathogen, can breach its normal habitat, resulting in life-threatening infections. *S. aureus* infections are of additional concern for populations with impaired immune function such as those with cystic fibrosis (CF) or chronic granulomatous disease. Multi-drug resistance is increasingly common in *S. aureus* infections, creating an urgent need for new antimicrobials or compounds that improve efficacy of currently available antibiotics. *S. aureus* biofilms, such as those found in the lungs of people with CF and in soft tissue infections, are notoriously recalcitrant to antimicrobial treatment due to the characteristic metabolic differences associated with a sessile mode of growth. In this work, we show that another CF pathogen, *Burkholderia cenocepacia*, produces one or more secreted compounds that can prevent *S. aureus* biofilm formation and inhibit existing *S. aureus* biofilms. The *B. cenocepacia*-mediated antagonistic activity is restricted to *S. aureus* species and perhaps some other staphylococci; however, this inhibition does not necessarily extend to other Gram-positive species. This inhibitory activity is due to death of *S. aureus* through a contact-independent mechanism, potentially mediated through the siderophore pyochelin and perhaps additional compounds. This works paves the way to better understanding of interactions between these two bacterial pathogens.

**IMPORTANCE:**

*Staphylococcus aureus* is a major nosocomial pathogen responsible for infecting thousands of people each year. Some strains are becoming increasingly resistant to antimicrobials, and consequently new treatments must be sought. This paper describes the characterization of one or more compounds capable of inhibiting *S. aureus* biofilm formation and may potentially lead to development of a new therapeutic.

## INTRODUCTION

*Staphylococcus aureus* is a commensal organism permanently and asymptomatically colonizing the anterior nares of 20%–40% of the general population and transiently colonizing an additional 20%–30%. *S. aureus* is also an opportunistic pathogen associated with infections of soft tissues, indwelling devices, and blood, as well as osteomyelitis, endocarditis, and pneumonia (1–6). *S. aureus* has a propensity to form biofilms, which result in persistent and recurrent infections (7). Antibiotics have a reduced effect against biofilm-associated organisms as the polymeric matrix surrounding the biofilm reduces the organism’s exposure to antimicrobials. In addition to providing a protective barrier, biofilm-associated cells have reduced metabolic activity and growth. Unfortunately, the majority of antibiotics used to treat *S. aureus* target cellular processes related to cell growth. Therefore, biofilm-associated *S. aureus* is inherently resistant to these antibiotics as biofilm-associated cells are typically metabolically reduced (8). Additionally, antibiotic resistance is increasing in clinical isolates of *S. aureus*, especially to methicillin and oxacillin, daptomycin, and the former drug of last resort, vancomycin (9).

*S. aureus* is one of the main pathogens that colonize the lungs of individuals with the genetic disorder cystic fibrosis (CF). Mutations in the *CFTR* gene that lead to cystic fibrosis manifest themselves phenotypically in the thickening of mucus on the surface of lung epithelial cells as well as immunological defects. The accumulation of mucus provides nutrients for colonization of opportunistic pathogens and inhibits many of the lung’s innate immune defenses, such as mucociliary clearance, resulting in persistent and recurrent bacterial infections for people with CF. The lungs of individuals with CF are often colonized by *S. aureus* strains, particularly early in childhood. This species is thought to form biofilms inside the host’s lungs but whether these biofilms are attached to the lung epithelia or as multicellular aggregates remains unclear (10, 11). *S. aureus* colonization frequently coincides with the presence of other major opportunistic pathogens, including *Pseudomonas aeruginosa* and *Burkholderia cenocepacia*, both of which are more commonly found in adults with CF, rather than in children (12). Few studies have examined the roles of CF pathogens together *in vitro* or *in vivo*, particularly in co-culture biofilms.

*B. cenocepacia* is a member of the *Burkholderia cepacia* complex (Bcc), a group of more than 20 closely related species. Members of this group, especially *B. cenocepacia* and *B. multivorans*, are opportunistic pathogens that infect the airways of people with CF and other immunocompromised individuals. In some cases, infection by Bcc members leads to rapid decline in lung function and “cepacia syndrome,” a fatal necrotizing pneumonia and septicemia (13, 14). The Bcc members are known for their large, plastic genomes and their ubiquity in many natural environments, including soils, sediments, and natural waters (15). Often, end-stage disease in CF is characterized by a strong reduction in the overall diversity in the CF lung, especially those that contain pathogens like *B. cenocepacia*, a phenomenon, which might imply direct mechanisms to outcompete or eliminate their competitors are employed by some bacterial species (16–19). This then introduces the question—How are they killing their competitors, and can we utilize this as new compounds to fight infections?

We sought to examine the potential mechanism(s) that underlies the clinical phenomenon in which *B. cenocepacia* can reduce or eliminate other bacteria from the lungs of CF individuals. For this, we assessed the ability of *B. cenocepacia* J2315 (an epidemic isolate) and other *B. cenocepacia* strains to abrogate or eliminate *S. aureus* strains in biofilms. We conducted experiments to study the potential mechanism(s) underlying this antagonistic relationship using the information from clinical phenomena and to better understand bacterial population dynamics as well as the selective forces that may influence the identification of novel therapeutics or innovative treatment strategies.

## MATERIALS AND METHODS

### Bioinformatics analyses

Clinical data from patients with CF in the U.S. were obtained in 2017 from the Cystic Fibrosis Foundation for 1 January 2010 to 31 December 2015 for 29 different fungal or bacterial pathogens (*Staphylococcus aureus* [MRSA and MSSA], *Haemophilus influenzae*, *Pseudomonas aeruginosa*, *B. cenocepacia*, *B. multivorans*, *B. cepacia*, *Burkholderia stabilis*, *Burkholderia vietnamiensis*, *Burkholderia dolosa*, *Burkholderia anthina*, *Burkholderia ambifaria*, *Burkholderia pyrrocinia*, *Burkholderia ubonensis*, *Burkholderia arboris*, *Burkholderia latens*, *Burkholderia lata*, *Burkholderia metallica*, *Burkholderia seminalis*, *Burkholderia contaminans*, *Burkholderia diffusa*, *any other Burkholderia complex species*, *Stenotrophomonas*, *Acinetobacter baumannii*, *Klebsiella pneumoniae*, *Serratia marcescens*, *Streptococcus milleri*, *Aspergillus*, *Candida*, and *Mycobacterium tuberculosis*). Specific health information was also obtained but not used in this study, and no identifying information was obtained. Patients that did not culture positive for any of the 29 requested pathogens were removed from our database. The request for third-party clinical data acquisition was considered as exempt by the University of Louisville Institutional Review Board.

The resultant data set contains 45,262–56,792 CF individuals with *S. aureus*, 5,832–6,463 CF individuals with *H. influenzae*, 33,335–36,306 CF individuals with *P. aeruginosa*, 1,926–2,252 CF individuals with Bcc members, 6,309–7,167 patients with *Stenotrophomonas* species, 314–401 CF individuals with *A. baumanii*, 458–583 CF individuals with *K. pneumoniae*, 687–799 with *S. marcescens*, 93–184 CF individuals with *S. milleri*, 6,306–7,069 CF individuals with *Aspergillus* species, and 6,410–9085 CF individuals with *Candida* species depending on the year. The R package *co-occur* (20, 21) was used with default parameters to assess the likelihood of the presence of two species in a given individual at a greater than, less than, or equal to random chance given the prevalence of these organisms in the CF population. This program analyzes the frequency and distribution of pair-wise sets of species in a data set and returns probabilities of high/neutral/low likelihood values for co-occurrence of the two species in samples.

### Bacterial strains and growth conditions

Bacterial strains used in this study are listed in Table 1. All cultures were maintained routinely in LB (Lennox formulation). Planktonic cultures were grown at 37°C with shaking unless otherwise noted.

**TABLE 1:** Strains used in this study

Strain	Features	Source	Reference
Gram-positive organisms			
*S. aureus* NRS77	Lab strain	Antimicrobial Resistance in *Staphylococcus aureus* (NARSA) program	Supported under NIAID/NIH Contract #272200700055C
*S. aureus* CHB06091	CF isolate	Boston Children’s Hospital	This study
*S. aureus* LAC	MRSA clinical isolate	Joanna Goldberg, Emory University	This study
*S. aureus* MW2	MRSA clinical isolate	Joanna Goldberg, Emory University	This study
*S. aureus* EUH001	MRSA clinical isolate	Joanna Goldberg, Emory University	This study
*S. aureus* EUH002	MRSA clinical isolate	Joanna Goldberg, Emory University	This study
*S. aureus* EUH003	MRSA clinical isolate	Joanna Goldberg, Emory University	This study
*S. aureus* EUH004	MRSA clinical isolate	Joanna Goldberg, Emory University	This study
*S. aureus* EUH005	MRSA clinical isolate	Joanna Goldberg, Emory University	This study
*S. aureus* T28260	MSSA CF isolate	David Greenberg, UT Southwestern Hospital	This study
*S. aureus* NRS 100	MRSA lab isolate	NARSA	
*S. aureus* NRS 299	MRSA isolate	NARSA	
*S. aureus* NRS 237	MRSA isolate	NARSA	
*S. aureus* 96773	MSSA CF isolate	Alan Junkins, Norton Hospital, Louisville, KY	This study
*S. aureus* 96779	MSSA CF isolate	Alan Junkins, Norton Hospital, Louisville, KY	This study
*S. aureus* 100165	MSSA CF isolate	Alan Junkins, Norton Hospital, Louisville, KY	This study
*S. aureus* 100619	MSSA CF isolate	Alan Junkins, Norton Hospital, Louisville, KY	This study
*S. aureus* 100662	MSSA CF isolate	Alan Junkins, Norton Hospital, Louisville, KY	This study
*S. aureus* 100619	MSSA CF isolate	Alan Junkins, Norton Hospital, Louisville, KY	This study
*S. aureus* 96635	MSSA CF isolate	Alan Junkins, Norton Hospital, Louisville, KY	This study
*S. epidermidis* NRS101	MSSA isolate	NARSA	
*Streptococcus saprophyticus* TJB031	Clinical isolate	Alan Junkins, Norton Hospital, Louisville, KY	This study
*Streptococcus warneri* TJB032	Clinical isolate	Alan Junkins, Norton Hospital, Louisville, KY	This study
*Enterococcus avium* TJB018	Clinical isolate	Alan Junkins, Norton Hospital, Louisville, KY	This study
*Enterococcus faecalis* TJB019	Clinical isolate	Alan Junkins, Norton Hospital, Louisville, KY	This study
*Enterococcus faecalis* TJB020	Clinical isolate	Alan Junkins, Norton Hospital, Louisville, KY	This study
*Bacillus cereus* TJB021	Clinical isolate	Alan Junkins, Norton Hospital, Louisville, KY	This study
*Bacillus cereus* TJB022	Clinical isolate	Alan Junkins, Norton Hospital, Louisville, KY	This study
*Bacillus* sp. TJB023	Clinical isolate	Alan Junkins, Norton Hospital, Louisville, KY	This study
*Bacillus* sp. TJB024	Clinical isolate	Alan Junkins, Norton Hospital, Louisville, KY	This study
*Bacillus* sp. TJB025	Clinical isolate	Alan Junkins, Norton Hospital, Louisville, KY	This study
*Listeria monocytogenes* TJB029	Clinical isolate	Alan Junkins, Norton Hospital, Louisville, KY	This study
*S. aureus* AH2547	GFP-expressing strain	Alexander Horswill, University of Colorado School of Medicine	(22)
*Burkholderia* strains			
*B. cenocepacia* J2315	Epidemic CF isolate, ET12 lineage	John LiPuma, Univ Michigan	(23, 24)
*B. cenocepacia* J2315 Δ*hppD*	Melanin-deficient strain	Joanna Goldberg, Emory Univ	(25)
*B. cenocepacia* HI2424	Soil isolate	John LiPuma, Univ Michigan	(26)
*B. cenocepacia* H111	CF isolate	Leo Eberl, University of Zurich	(27)
*B. cenocepacia* K56-2	Epidemic CF isolate, ET12 lineage	John LiPuma, Univ Michigan	(28)
*B. cenocepacia* C5424	Clinical isolate	Joanna Goldberg, Emory Univ	(29)
*B. orbicola* AU1054	Epidemic CF isolate, PHDC lineage	John LiPuma, Univ Michigan	(30)
*B. orbicola* MC0-3	Corn rhizosphere isolate	James Tiedje, Michigan State Univ	(24, 31)
*B. dolosa* AU0158	Epidemic CF isolate	Greg Priebe, Boston Children’s Hospital	(32)
*B. multivorans* CF1	CF isolate	Joanna Goldberg, Emory Univ	(33)
*B. multivorans* CGD1	CF isolate	Joanna Goldberg, Emory Univ	(33)
*B. pseudomallei* Bp82	BSL2 Δ*purM* strain	Herbert Schweizer, UFlorida	(34)
*B. cenocepacia* H111 Δ*orbJ*	Does not produce ornibactin	Leo Eberl, University of Zurich	(35)
*B. cenocepacia* H111 Δ*pchAB*	Does not produce pyochelin	Leo Eberl, University of Zurich	(35)
*B. cenocepacia* H111 Δ*orbJ* Δ*pchAB*	Does not produce ornibactin or pyochelin	Leo Eberl, University of Zurich	(35)

### Biofilm survival assays

Approximately 10^5^ mid-log phase bacterial cells were inoculated into triplicate wells of mono- and co-culture conditions in Tryptic Soy Broth (TSB) in 96-well round-bottomed polyvinyl chloride (PVC) plates (VWR #29442–384) in a final volume of 100 µL. In later assays, the LB was supplemented with 1% glucose and 150 mM MOPS buffer (pH 7.0), instead of TSB, for biofilm assays unless otherwise noted. Co-culture wells contained 2 × 10^5^ cells total in the same volume. We note here that similar results were obtained when co-culture wells contained 10^5^ cells total (i.e., 5 × 10^4^ cells for each species). Plates were sealed with porous rayon film (VWR, 60941–086) and incubated at 37°C in humidity chamber with 2-cm sterile water below the plates for 1, 3, or 7 days. At each time point, the rayon film was removed carefully to avoid aerosolization and splashing, wells were washed twice with sterile water to remove planktonic cells, and 150 µL of recovery medium (TSB +1% Tween 20 [vol/vol]) was added. Plates were sealed with pierceable aluminum foil (VWR, 75805–268) and sonicated at 40 kHz for 10 min in a Bronson 3200 water bath sonicator for routine lifting of cells. The use of PVC plates and the sonication settings were optimized for *B. cenocepacia* and *S. aureus* prior to commencing the co-culture survival assays (Fig. S1). Immediately after sonication, cultures were serially diluted 10-fold in sterile 96-well polystyrene plates and plated onto LB agar via 10-µL drip dilutions. Plates were incubated 24–48 h at 37°C, and the number of colony-forming units (CFUs) was recorded. Manual counts of the colonies were performed utilizing phenotype to distinguish the identity of co-occurring partners. In pilot experiments, manual counts did not differ when using selective agar for each of the species compared to when plated together. Thus, mixed cultures were routinely plated together for the experiments described in this work.

### Optimization experiments for the growth medium and pH determination experiments

*B. cenocepacia* and *S. aureus* were inoculated in mono- or co-culture and incubated over the course of 10 days to examine the pH of the biofilm conditions. On each day, the pH was taken for each well using a probe pH meter. and the biofilms were covered with rayon film again and incubated again until the next day.

For medium optimization experiments, multiple media used for inoculating *B. cenocepacia* and *S. aureus* were used. Plain TSB was compared to LB Lennox broth and were prepared according to the manufacturer’s instructions. SCFM and M9 +0.5% (wt/vol) casamino acid media were prepared from filter-sterilized stocks of individual components within 2 weeks of their use. In a subsequent set of assays, LB (Lennox) broth ± 1% glucose was compared to TSB ± 1% glucose and to either media without supplementation. When altering the carbohydrates, LB (Lennox) broth + 1% glucose, 1% galactose, 1% lactose, or 1% sucrose was added to the medium prior to autoclaving. In the final optimization experiments, LB (Lennox) broth +100 or 150 mM MOPS was generated, and differing concentrations of glucose were added to separate aliquots of each buffered medium prior to autoclaving. Biofilm survival assays were then performed in these different media as described above.

### Generation of cell-free supernatants and heat-killed overnight cells

*B. cenocepacia* wild-type or mutant strains were inoculated into TSB at 10^6^ CFU/mL and incubated at 37°C for 3 days as described above. After 3 days, the biofilm supernatants were removed via a micropipette, centrifuged at 21,130 × *g* for 1 min, and filter-sterilized through a 0.45-µm syringe filter (VWR #28145–505). Alternatively, supernatants from overnight cultures of *B. cenocepacia* J2315 were collected, centrifuged, and filter sterilized as stated above. For overnight cell preparations, the *B. cenocepacia* cell pellets resulting from centrifugation following overnight incubation were also resuspended in TSB, diluted 1:100 in TSB, heated in a 65°C water bath for 30 min, and then sonicated at 60% amplitude for 10-s pulses over a period of 10 min at 20 kHz in a Qsonica cup ultrasonicator (Qsonica, Q800R). Cell death was verified by a lack of growth on LB agar plates. *S. aureus* was then inoculated into each of these supernatants or killed cell samples at 10^6^ CFU/mL. One hundred microliters of suspension was added into three to five replicate wells of a 96-well plate, sealed with breathable rayon film, and incubated for 1, 3, or 7 days at 37°C in a humidity chamber as indicated. Survival of *S. aureus* in each condition was quantified as described above.

### Planktonic- and biofilm-associated survival during co-inoculations

*B. cenocepacia* and *S. aureus* mono- and co-culture biofilms were generated using the methods described above. At 7 days post-inoculation, the liquid above each biofilm (i.e., the planktonic cells) were removed with micropipettors, serially diluted, and plated using the drip dilution method. Biofilms for each well were washed, put in recovery medium, sonicated, serially diluted, and plated as described above. *B. cenocepacia* and *S. aureus* colonies were quantitated for each condition.

### Microscopy and fluorescence quantitation

Twenty-four replicate wells of *B. cenocepacia* J2315 biofilms were established as described above and incubated for 7 days. The supernatants were removed and centrifuged for 5 min at 15,000 rpm in a microcentrifuge. Supernatants were then filter sterilized using a syringe attached to a 0.22-µm syringe filter. *S. aureus* AH2547, bearing the pCM29 plasmid, which expresses GFP, were grown in LB + 1% glucose + 150 mM MOPS (hereafter known as LB++) + 10 µg/mL of chloramphenicol overnight, diluted 1:50, and allowed to grow to mid-log phase. *S. aureus* cells were diluted to 10^7^ CFU/mL in either LB + 1% glucose +1 50 mM MOPS, *B. cenocepacia* 7-day biofilm supernatant, or 1× sterile PBS. At least four replicate wells of PVC plates were inoculated with 100 µL of each mixture and incubated as described above for biofilms. After 24 and 72 h, one plate was harvested by removing the rayon film, collecting individual supernatants, and placing in microfuge tubes (planktonic cells). Biofilms from each well were collected individually as described above. Biofilm and planktonic cells were centrifuged for 5 min at 15,000 rpm in a microcentrifuge and the supernatants discarded. Cells were resuspended in 20 µL of 1× PBS + 10% propidium iodide (Tonbo Sciences, #13–6990-T200). Samples were quantitated for absorbance at 280 nm (“total protein” surrogate), GFP fluorescence, and propidium iodide fluorescence on a Nanodrop 2000. Ten microliters of each sample was also imaged by confocal microscopy using a Nikon Eclipse Ti microscope with both the 60× and 100× objectives. The GFP (487 nm) and TxRed (561 nm) lasers were used for excitation wavelengths for GFP and propidium iodide respectively, and DIC images were also captured. The power and gain for each laser and DIC for each sample were optimized to show maximum fluorescence without saturation of the pixels.

### Metabolomics

Seven-day-old biofilms of *B. cenocepacia* and *S. aureus* strains were generated as described above in LB ++medium. Supernatants from at least 10 wells were collected and frozen at −80°C for 2 h or overnight, then freeze dried in a LabConco 2.5L Benchtop Freeze Dryer. Samples were stored at 4°C until submission to the Center for Regulatory and Environmental Analytical Metabolomics at the University of Louisville. Four biological replicates for each sample were analyzed separately by 2D LC-MS after resuspension in 50% acetonitrile. Metabolites were mapped using a suite of databases though Compound Discoverer v3.1 software: *E. coli* Metabolome Database, Fecal Metabolome database, KEGG, Saliva Metabolome Database, Urine Metabolome Database, Compound Classes/Therapeutics/Prescription Drugs (including only the Endogenous Metabolites, Natural Products/Medicines, Natural Toxins, Small Molecule Chemicals, Steroids/Vitamins/Hormones, and Therapeutics/Prescription Drugs groups). The raw data have been uploaded to Figshare and can be accessed here: https://doi.org/10.6084/m9.figshare.28543700.v1.

### *S. aureus* biofilm survival in the presence of iron and metabolites

*S. aureus* cultures were grown overnight in 5 mL of LB medium at 37°C with shaking. Cultures were diluted 1:50 in fresh LB medium and allowed to grow at 37°C with shaking until the OD_600_ reached ~0.8–1.0. Cultures were diluted 10^6^ CFU/mL in LB++ containing different concentrations of N-acetyl-DL-valine (Ambeed), uric acid (Ambeed), DL-α-aminoadipic acid (Ambeed), L(−)-carnitine (Ambeed), N-acetylmuramic acid (Bachem Americas Inc), 4-methoxybenzylisothiocyanate (Ambeed), or urea (BeanTown Chemical). In separate experiments, *B. cenocepacia* cultures were diluted into M9 +1% casamino acids + 1% glucose with or without differing concentrations of iron(III) chloride hexahydrate (Thermo Scientific Chemicals) purchased through VWR. Replicate wells of a 96-well PVC were inoculated with 100 μL of each sample, sealed with rayon film, and incubated at 37°C in a humidity chamber for 3 days. Biofilms were washed, sonicated, and plated as described above.

### Statistical analysis

Student’s *t*-tests and analyses of variance (ANOVA s) were used to compare the survival of bacterial strains in co-culture compared to mono-cultures of the same species. Use of a statistical test was dictated by the biological questions being asked of the data. When values were beneath the limit of detection (100 CFU/mL), the values of 99, 100, and 101 were used for statistical purposes only. Statistical analyses were performed using GraphPad Prism v. 5.0.1.

## RESULTS

### Bioinformatic analysis of CF lung microbiology

**Fig 1:**
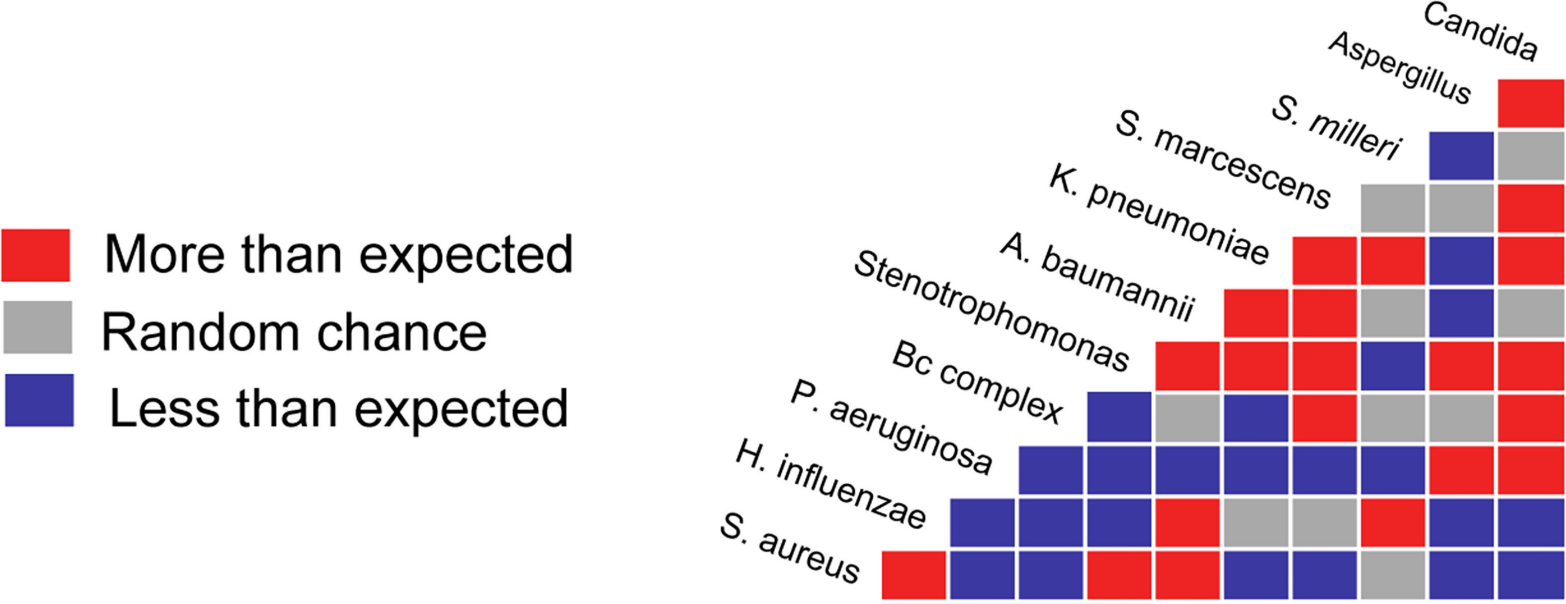
Co-occurrence analysis of microbiological data from individuals with CF. Patient culture data spanning the years 2010–2015 was analyzed using the Probabilistic Species Co-Occurrence Analysis package for R statistical software. The data from all years were analyzed together. Red boxes represent co-cultures from the same CF lung present more often than would be expected by random chance. Blue boxes represent co-culture presence at a rate less than expected by random chance. Gray boxes represent those co-cultures present at rates expected by random chance.

Microbiological data from CF patients in the U.S. over the course of 6 years was obtained from the Cystic Fibrosis Foundation and analyzed for patterns in co-colonization between our pathogens of interest. We observed largely antagonistic interactions between the five most common CF pathogens, *S. aureus*, *Haemophilus influenzae*, *P. aeruginosa*, Bcc members, and *Stenotrophomonas* sp. (Fig. 1). One exception was the positive interaction between *S. aureus* and *H. influenzae*, both of which are commonly found in children with CF. In less common pathogens, such as *Acinetobacter baumanii*, *Klebsiella pneumoniae*, *Serratia marcescens*, *Streptococcus milleri*, or in the fungal pathogens *Candida albicans* and *Aspergillus fumigatus*, interactions varied based on year, presumably due to lower statistical robustness (i.e., fewer reported cases), while the interactions for common pathogens did not.

While many of these *in vivo* co-culture observations were interesting, we chose to focus on one interaction, the negative interaction between *S. aureus* and *B. cenocepacia*, for follow-up studies. This is because clinical data from CF patients have shown that diversity decreases at end-stage disease, particularly when *Burkholderia* species are present (17, 36). *B. cenocepacia* generally grows much slower than *S. aureus in vitro*. Therefore, this could imply that *B. cenocepacia* exert its inhibition through a direct mechanism rather than outcompeting *S. aureus* for nutrients.

### *In vitro* analysis of *S. aureus* and *B. cenocepacia* co-culture biofilms

First, the substrate used to grow biofilms and biofilm removal methods were optimized to ensure adequate conditions for both organisms were used. Both the time that biofilms were sonicated to lift them from the plates and the types of plastic used were optimized for the interacting partners. From this, 10 min in the sonicator bath was sufficient to maximally lift both *B. cenocepacia* and *S. aureus* from the plate. In addition, polyvinylchloride plates were shown to be more effective at growing both *S. aureus* and *B. cenocepacia* under these conditions.

**Fig 2:**
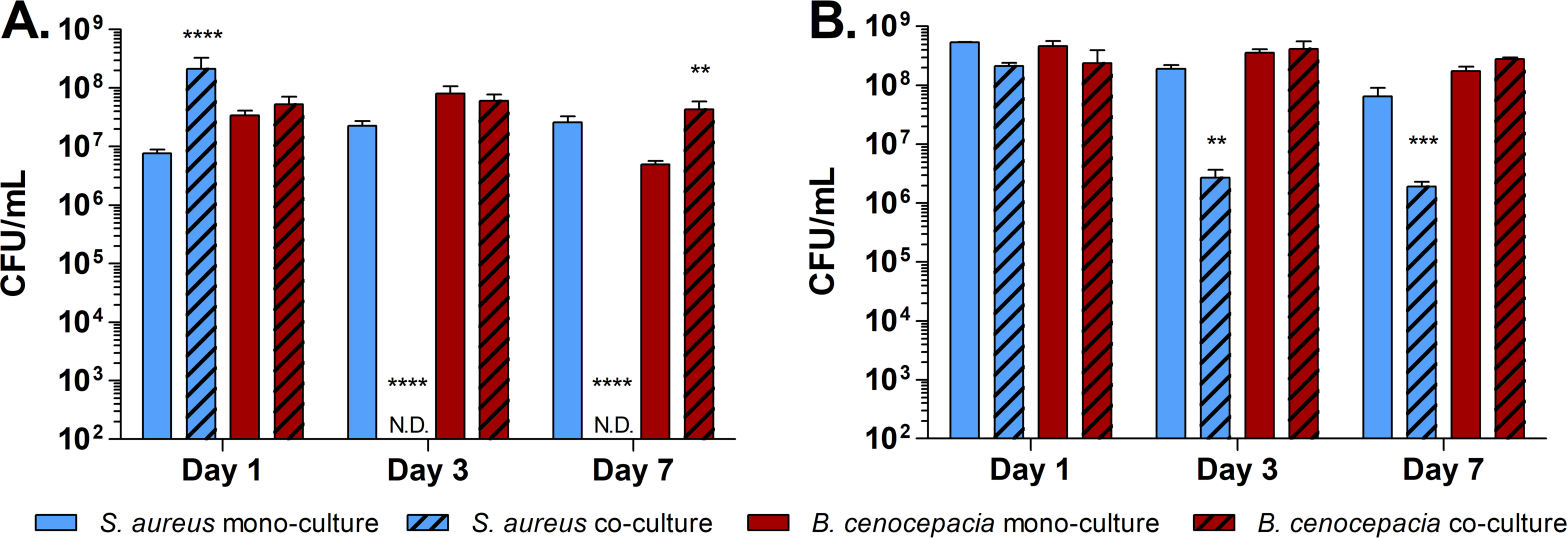
*B. cenocepacia* J2315 inhibits *S. aureus* NRS77 biofilm formation and maintenance. Viable cell counts from washed and sonicated biofilms over time when (**A**) *B. cenocepacia* and *S. aureus* were inoculated simultaneously, or (**B**) *B. cenocepacia* was added to established 24-h *S*. *aureus* biofilms. Viable cell counts are shown in solid or striped bars for mono- and co-cultures, respectively. N.D. indicates bacterial concentrations less than the limit of detection (dashed lines). Error bars indicate one standard deviation of the data from three biological replicates. Student’s *t*-tests indicate significant differences between co-culture and cognate mono-culture biofilm formation (** *P* < 0.01, ***P < 0.001, ****P < 0.0001).

To assess whether *B. cenocepacia* can inhibit *S. aureus* biofilms *in vitro*, the ability of *S. aureus* and *B. cenocepacia* to survive in biofilms was measured over 1-, 3-, and 7-days post-inoculation using *in vitro* conditions. The survival of these strains in co-culture was compared to mono-cultures of each strain conducted in parallel. In 1-day-old biofilms, *S. aureus* and *B. cenocepacia* in co-culture are found at levels similar or higher than cognate mono-cultures (Fig. 2A). However, by days 3 and 7, *S. aureus* in co-culture is undetectable, while viable *S. aureus* in mono-culture remains at high levels. *B. cenocepacia* remained largely unaffected by the presence of *S. aureus* over time in mono-culture (one-way ANOVA *P* = 0.0581) and in fact was slightly higher in co-culture at day 7.

We further investigated whether *B. cenocepacia* could inhibit existing *S. aureus* biofilms. Analysis of *S. aureus* survival after *B. cenocepacia* was added to 1-day-old *S. aureus* biofilms indicates a significant reduction of *S. aureus* in biofilm by 3 days after *B. cenocepacia* was added (Fig. 2B) but not to undetectable levels as is observed when these strains are inoculated concomitantly. Together, these data demonstrate that *B. cenocepacia* is capable of inhibiting *S. aureus* biofilm formation but is not as effective at reducing *S. aureus* within already established biofilms.

### Conservation of *B. cenocepacia* antagonistic effects

**Fig 3:**
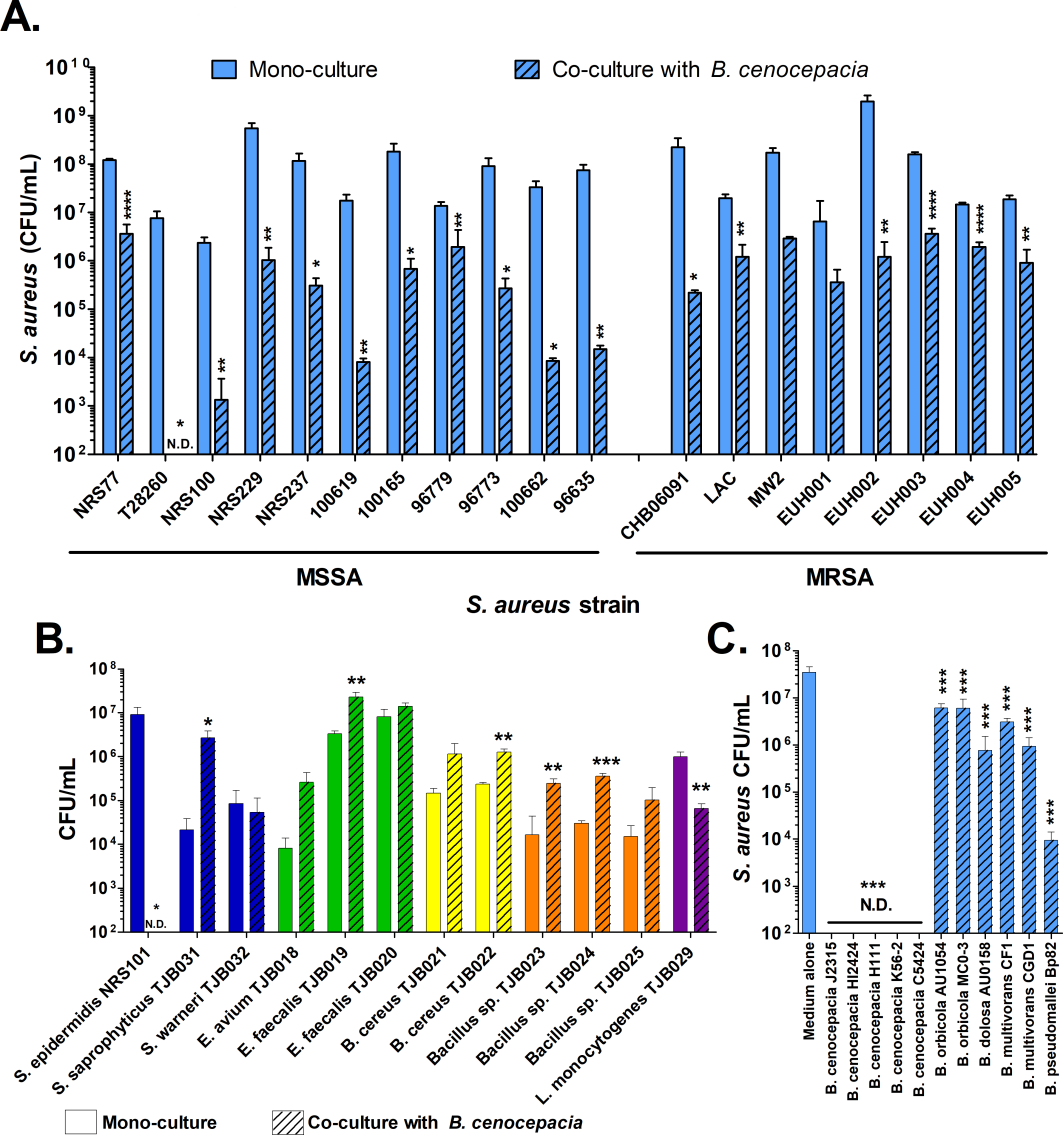
*S. aureus* biofilm inhibition by *B. cenocepacia* is conserved within the species. (**A**) Various *S. aureus* strains were inoculated with *B. cenocepacia* J2315, and survival was measured. (**B**) *B. cenocepacia* J2315 was co-inoculated with various clinical *Staphylococcus* strains and other Gram-positive species/strains. For panels (A) and (B), *t*-tests indicate significant differences between co-culture and cognate mono-culture biofilm formation. (**C**) *S. aureus* NRS77 was inoculated with various *Burkholderia* strains. For panel (C), one-way ANOVA analysis using Dunnett’s post-test with all test strains compared to medium alone control was assessed. For all panels, viable cell counts are shown in solid or striped bars for mono- and co-cultures, respectively, from 7-day-old biofilms. N.D. indicates bacterial concentrations less than the limit of detection (dashed lines). Error bars indicate 1 standard deviation of the data from three biological replicates. * *P* <0.05,** *P* < 0.01, *** *P* < 0.001, **** *P* < 0.0001.

To determine whether the observed antagonism between *S. aureus* NRS77 and *B. cenocepacia* J2315 is specific to the *S. aureus* NRS77 isolate used in our initial studies, the effect of *B. cenocepacia* J2315 on additional *S. aureus* clinical strains was tested. These strains were obtained from burn patients, CF patients, patients with bacteriemia, and eye infections, from multiple hospitals (Table 1). All methicillin-sensitive *S. aureus* (MSSA) were significantly reduced, some to below detectable levels, in co-culture with *B. cenocepacia* J2315 compared to the cognate *S. aureus* mono-cultures (Fig. 3A). All methicillin-resistant *S. aureus* (MRSA) strains, with the exception of two, were significantly reduced in co-culture versus mono-culture as well. However, the levels of inhibition for MRSA strains were less in general than that observed for MSSA strains, but this difference was not significant by *t*-test (*P* = 0.2154).

Other Gram-positive species were tested for their capacity to be inhibited by *B. cenocepacia*. As shown in Fig. 3B, only *Staphylococcus epidermidis* and *Listeria monocytogenes* were significantly inhibited in co-culture with *B. cenocepacia* compared to mono-culture conditions. Other *Staphylococcus*, *Enterococcus*, and *Bacillus* species were not inhibited and were, in fact, often found at significantly higher levels in co-culture with *B. cenocepacia*. This implies that *B. cenocepacia* can inhibit *S. aureus* and possibly closely related staphylococcal species, but its inhibitory activity is fairly limited in breadth.

To determine if the antagonistic effect imparted by *B. cenocepacia* J2315 is maintained at the strain, species, or genus level, several *B. cenocepacia* strains and other *Burkholderia* species were co-inoculated with *S. aureus* NRS77, and *S. aureus* biofilm-associated cell viability was observed 7 days post co-inoculation. All of the Bcc members significantly reduced *S. aureus* viability in our experiments (Fig. 3C). All *B. cenocepacia* strains reduced *S. aureus* to below the level of detection. A more modest, but still significant, antagonistic effect was observable for most other *Burkholderia* species. Interestingly, one of the isolates that inhibits *S. aureus*, *Burkholderia pseudomallei* Bp82, is more distantly related to *B. cenocepacia* than the other members of the Bcc, and yet. it yielded a high level of *S. aureus* inhibition (~ 4,000-fold) outside of *B. cenocepacia*. Due to the consistent and strong inhibition observed with *B. cenocepacia* strains, it seems likely that, at least within this species, the mechanism underlying the inhibition may be the similar, but that there may be other inhibitory mechanisms in other *Burkholderia* species.

### *B. cenocepacia* inhibition of *S. aureus* does not require cell-to-cell contact

**Fig 4:**
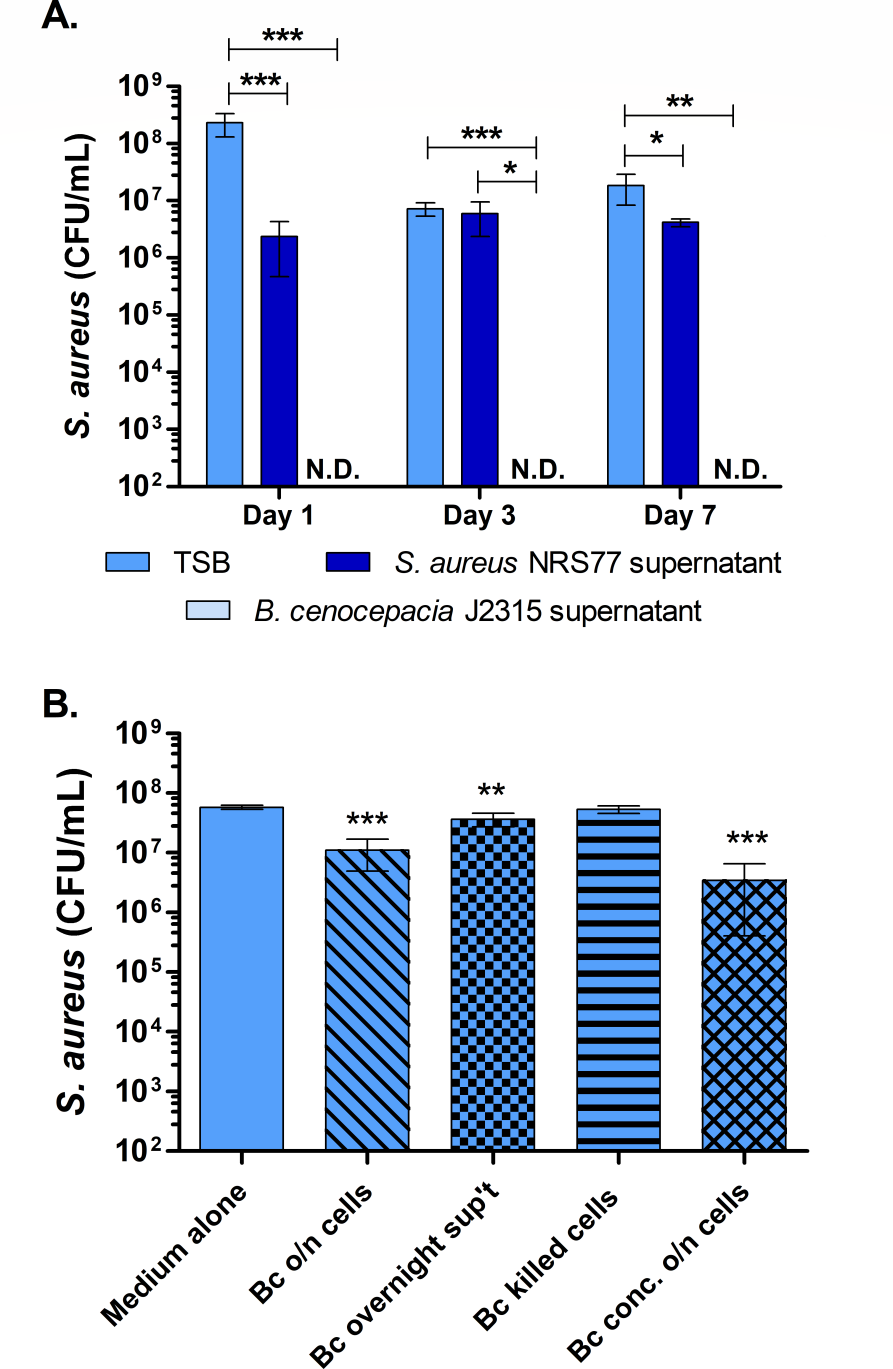
*S. aureu*s biofilm inhibition by *B. cenocepacia* is not mediated by cell-to-cell contact. (**A**) Biofilm-associated *S. aureus* NRS77 survival in TSB versus when inoculated into 3-day-old *S. aureus* sterile biofilm supernatant or 3-day-old *B. cenocepacia* J2315 sterile biofilm supernatant. (**B**) Biofilm-associated *S. aureus* survival in *B. cenocepacia* J2315 cells, *B. cenocepacia* J2315 cells from overnight cultures, the sterile supernatant from *B. cenocepacia* culture grown overnight with aeration, *B. cenocepacia* culture killed with high-frequency sonication, or concentrated *B. cenocepacia* cells. Experiments for panels (A) and (B) were conducted at the same time or separately with the same results. For both panels, viable cell counts were quantitated from 1- and 3-day-old biofilms. Error bars indicate one standard deviation of the data. Student’s *t*-tests indicate significant differences in CFU density between *S. aureus* alone and *S. aureus* in conditions containing *B. cenocepacia* cells or supernatant (*P < 0.05, ** *P*< 0.01, *** *P*< 0.001, **** *P*< 0.0001).

We next hypothesized that *B. cenocepacia* may produce a secreted compound that can reduce *S. aureus* in biofilms. To test this hypothesis, *S. aureus* was inoculated into supernatants collected from 3-day-old mono-culture biofilms of *B. cenocepacia* or *S. aureus*. While the *S. aureus* biofilm supernatant treatment resulted in *S. aureus* survival at levels comparable to TSB (especially by day 3), there was a complete inhibition of *S. aureus* inoculated into *B. cenocepacia* cell-free supernatants, even after only 1 day (Fig. 4A). The addition of *B. cenocepacia* supernatants from overnight cultures, concentrated *B. cenocepacia* cells from overnight cultures, or heat-killed *B. cenocepacia* did not result in similar inhibitory levels on *S. aureus* as the *B. cenocepacia* biofilm supernatants, though slight reductions in *S. aureus* survival occurred for conditions with living *B. cenocepacia* overnight cells or overnight supernatants (Fig. 4B). Further, the inhibitory effect of biofilm supernatants on *S. aureus* was also observed for many other *Burkholderia* species (Fig. S2). These data indicate that the antagonistic effect is potentially achieved by one or more products secreted by *Burkholderia* during biofilm growth.

### Effect of pigment and pH on *S. aureus* inhibition

One obvious mechanism by which *B. cenocepacia* could be inhibiting *S. aureus* would be through acidifying the medium. We tested the pH of the mono-cultures and co-cultures over time to determine if pH played a role in mediating this inhibition. By day 1, *S. aureus* in mono-culture had reduced the pH of the biofilm supernatant to a pH ~5.97, but the pH increased to 8.2 slowly over 6 days (Fig. S3). *B. cenocepacia* mono-culture was closer to the medium alone control at a pH ~7.2 at day 1 but rose to pH >8.0 after 3 days. The mixed culture started at a pH of 6.3 and rose quickly to >8.0 by day 4. Therefore, the pH within the co-culture conditions lies between the *B. cenocepacia* and *S. aureus* co-culture conditions and was only significantly different from the *S. aureus* mono-culture at day 3 based on two-way ANOVA analysis (*P* < 0.001). Thus, the acidification of the medium does not seem to be the mechanism of action for the *B. cenocepacia* antagonism of *S. aureus* in biofilms; however, the rapid rise in pH in the mixed biofilms may have altered the *S. aureus* biofilm to make it more susceptible to inhibition by *B. cenocepacia*—a possibility that cannot be ruled out at this time.

We anecdotally observed that darker wells of our 96-well plate tended to show more *S. aureus* inhibition suggesting that pyomelanin, the brown pigment produced by *B. cenocepacia*, may be playing a role in this inhibition. We obtained a pyomelanin-deficient strain of *B. cenocepacia* that lacks the *hppD* gene (25). When co-inoculated, this mutant inhibited *S. aureus* at levels similar to wild-type *B. cenocepacia* J2315 (Fig. S3) suggesting that pyomelanin was not the cause of *S. aureus* inhibition. Further testing of *B. cenocepacia* mutants lacking additional metabolites, such as ornibactin, pyochelin, or others, remains to be explored.

### Optimization of conditions for maximal and reliable production of the inhibitory substance

We initially observed that the *B. cenocepacia* inhibition of *S. aureus* was robust and repeatable. After many of these experiments were completed, however, this inhibition became quite variable and eventually was not observed at all. The conditions that most consistently reproduced and maximized the *B. cenocepacia*-mediated inhibition of *S. aureus* were then explored. The water source (ultrapure versus tap water), the pH of the medium, the medium used, and the use of different carbohydrates (glucose, lactose, galactose, sucrose) were all investigated. The use of ultrapure versus tap water to reconstitute media made no difference in the levels of inhibition (data not shown). There was no inhibition with the use of other types of media such as M9 + 0.5% casamino acids or synthetic CF sputum medium (SCFM) (37) either (Fig. S4). We added different concentrations of glucose and found that increasing glucose improved *S. aureus* inhibition. However, at the highest levels of glucose, the medium acidified and *S. aureus* in mono-culture was strongly reduced. Therefore, to counteract the acidification, we also tested with several concentrations of MOPS buffer and found that using autoclaved LB (Lennox) broth made with ultrapure water and supplemented with 1% glucose and 150 mM MOPS (pH 7.0) led to the most robust and reproducible inhibition of *S. aureus* by *B. cenocepacia* (Fig. S4). From these results, it appears that the antagonistic interaction observed between these two organisms seems to be highly sensitive to environmental conditions, and this interaction appears to be highly dependent on the nutritional compounds and concentrations in the medium.

These conditions restored the inhibition reproducibility and were then used from that point forward. We note here that most of the experiments described earlier were repeated in the new medium, and we observed similar results for all of them.

### *B. cenocepacia* supernatants cause *S. aureus* death *in vitro*

**Fig 5:**
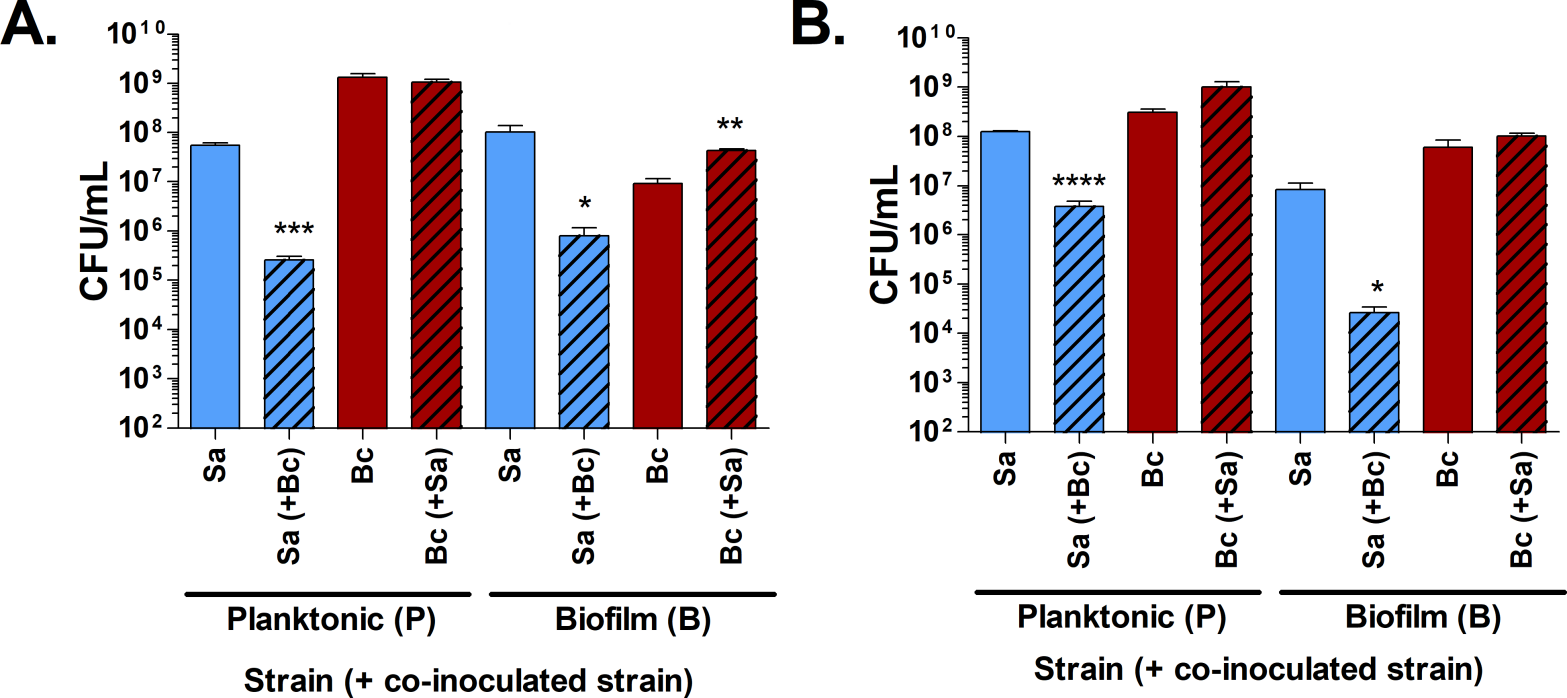
*S. aureus* NRS77 survival in planktonic or biofilm conditions in the presence or absence of *B. cenocepacia* J2315. Viable cell counts were quantitated from 7-day-old biofilms or the supernatant above the biofilms (planktonic cells) grown in LB + 1% glucose + 150 mM MOPS. Error bars indicate 1 standard deviation of the data. Student’s *t-*tests indicate significant differences in CFU density between a mono-culture control and its cognate co-culture (*P < 0.05, ****P < 0.0001).

We next sought to determine whether compound(s) secreted by *B. cenocepacia* killed *S. aureus* or simply prevented biofilm formation. We hypothesized that if *B. cenocepacia* only inhibits biofilm formation, then the concentration of planktonic *S. aureus* (i.e., non-biofilm-associated cells in the same well) would not change. To test this, *S. aureus* survival in biofilms and in planktonic supernatants was measured in response to co-inoculation with *B. cenocepacia* cells. As shown in Fig. 5, *B. cenocepacia* survived well in mono-culture and co-culture in both biofilms and planktonic cultures. Additionally, *S. aureus* survives well in mono-culture in both planktonic and biofilms after 7 days of incubation. However, it is inhibited in both planktonic and biofilm in the presence of *B. cenocepacia*, albeit not completely reduced beyond the limits of detection in this experiment, when the strains were co-inoculated. This implies that the *B. cenocepacia* inhibition of *S. aureus* is due to the death of both planktonic- and biofilm-associated cells rather than to only inhibition of biofilm formation or maintenance.

The results from the co-culturing experiment showed a mild inhibition of *S. aureus* in planktonic conditions when co-inoculated with *B. cenocepacia*. We further examined the fate of biofilm and planktonic *S. aureus* when exposed to *B. cenocepacia* J2315 biofilm supernatants. For this, *S. aureus* AH2547, which is tagged with GFP, was used as a marker for *S. aureus* cells. These cells were inoculated into fresh medium alone as a control or inoculated into *B. cenocepacia* 7-day-old biofilm supernatants that had been filtered to remove all *B. cenocepacia* cells. After 24 and 72 h, planktonic- and biofilm-associated *S. aureus* cells were mixed with propidium iodide, which is a large fluorescent DNA-binding dye that can only penetrate cells whose cell walls are compromised (i.e., dying or close to dying) and either quantitated using spectrophotometry or imaged using confocal microscopy. Using supernatants rather than live *B. cenocepacia* cells allowed for the visualization and quantitation of only *S. aureus* without interference from *B. cenocepacia* cells.

**Fig 6:**
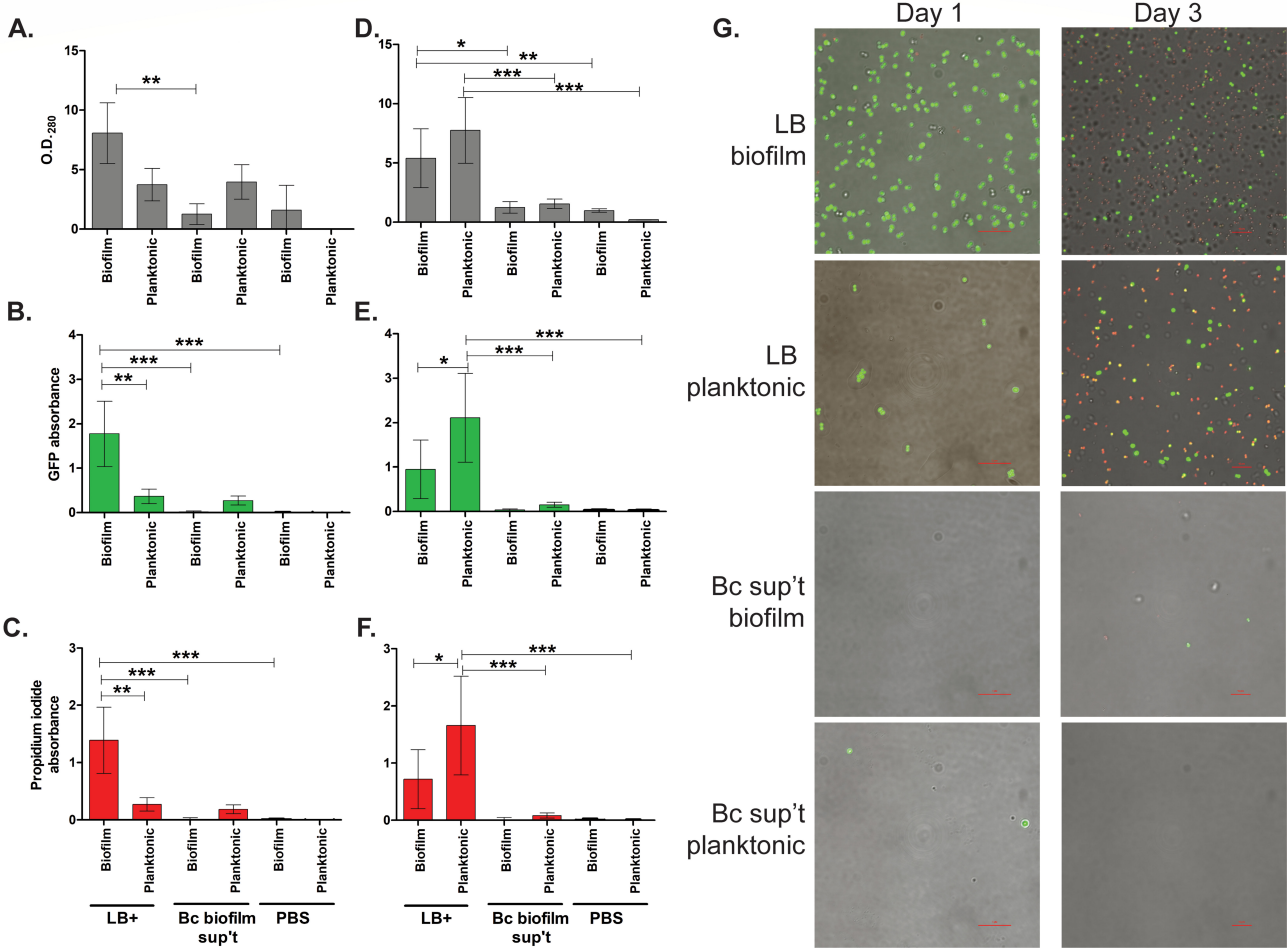
The fate of *S. aureus* grown in *B. cenocepacia* biofilm supernatants. (**A–F**) Green fluorescent *S. aureus* exposed to LB + 1% glucose + 15 mM MOPS (LB+) medium, *B. cenocepacia* 7-day-old biofilm supernatants, or 1× PBS was quantitated by spectrophotometry. (**A and D**) Total protein quantitated by absorbance at 280 nm. (**B and E**) GFP absorbance. (**C and F**) Propidium iodide absorbance. (**A–C**) Twenty-four hours after inoculation. (**D–F**) Seventy-two hours after inoculation. Error bars indicate 1 standard deviation of the data. One-way ANOVAs with Tukey’s post-test indicate significant differences between co-culture and cognate mono-culture biofilm formation (*P < 0.05. **P < 0.01, ***P < 0.001). (**G**) Representative merged images from confocal microscopy are shown. Day-1 images are shown at ×1,000, while day 3 images are shown at ×600 to show a greater width of field.

*S. aureus* grown in medium alone showed robust growth and GFP expression in both biofilm and planktonic cells when incubated for 24 h as expected (Fig. 6). By day 3, *S. aureus* showed an increase in propidium iodide, presumably as cells began to die after nutrients had been consumed. *S. aureus* exposed to *B. cenocepacia* 7-day-old biofilm supernatants showed virtually no cells after 24 or 72 h (Fig. 6; Fig. S6); however, a few living cells remain in planktonic conditions, but these represent both living and dying cells as both GFP and propidium iodide levels remain roughly similar. These data support our previous data in which *B. cenocepacia* biofilm supernatants robustly inhibit *S. aureus* survival (Fig. 4) and that *B. cenocepacia* biofilm supernatant is more potent than growth with live *B. cenocepacia* cells (Fig. 5). Further, this suggests that the inhibitory compound produced by *B. cenocepacia* during biofilm formation results in *S. aureus* lysis quickly as virtually no living *S. aureus* cells were observed in either planktonic- or biofilm-associated conditions.

### Potential mechanisms that underlie the *B. cenocepacia* inhibition of *S. aureus*

**Fig 7:**
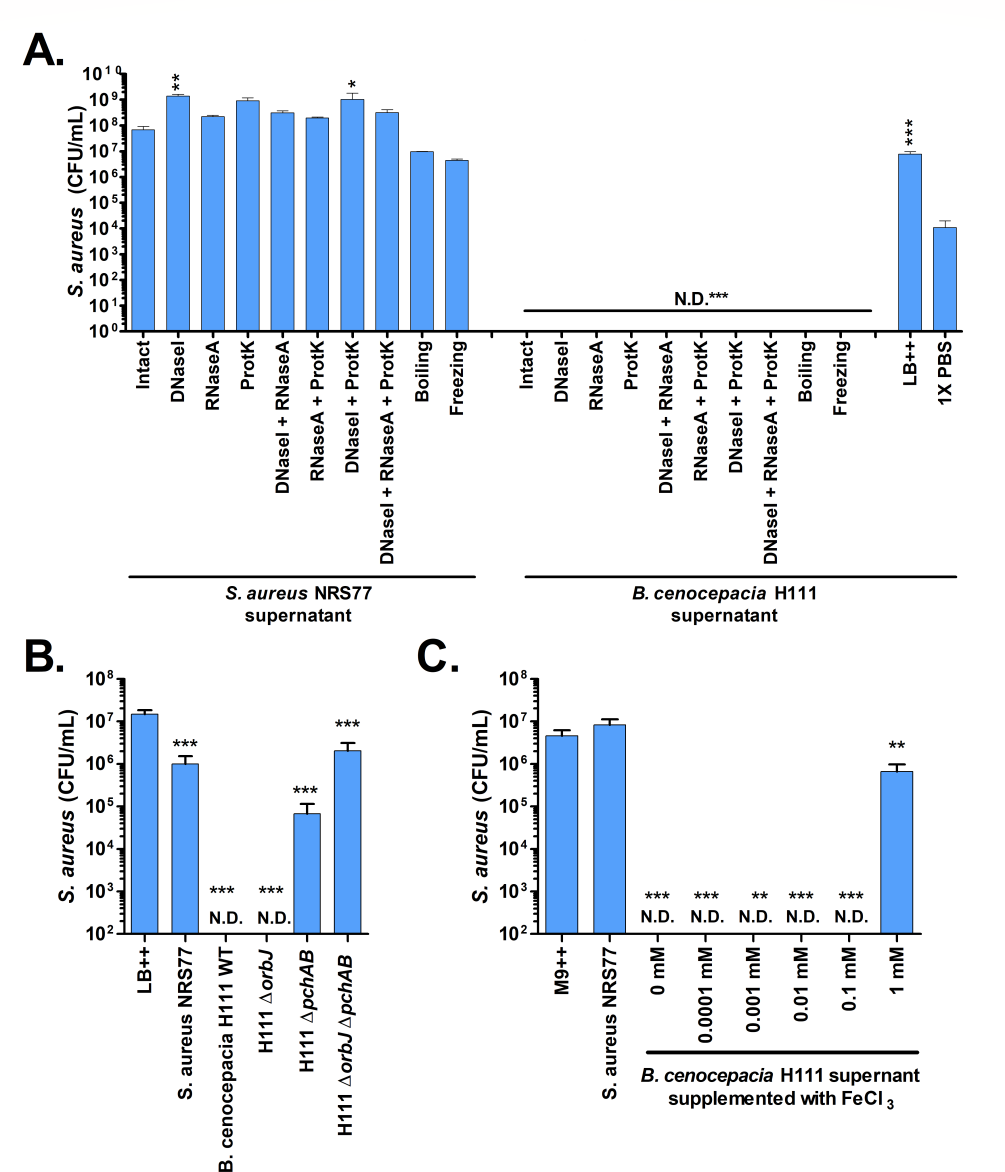
The role of pyochelin in the inhibition of *S. aureus* by *B. cenocepacia*. (**A**) *S. aureus* survival in 7-day-old biofilms generated with untreated (“intact”) or treated supernatants harvested from 7-day-old biofilms of *S. aureus* or *B. cenocepacia*. (**B**) *S. aureus* survival after 7 days of incubation with *B. cenocepacia* H111 wild-type (WT) or siderophore mutant supernatants. (**C**) *S. aureus* concentrations following incubation for 3 days under biofilm conditions after inoculation with *B. cenocepacia* supernatant supplemented with increasing amounts of Fe_3_Cl_2_. For all panels, LB ++ indicates LB (Lennox) supplemented with 1% glucose and 150 mM MOPS. N.D. indicates levels of *S. aureus* beneath the level of detection (100 CFU/mL). Asterisks indicate significant changes from controls based on one-way ANOVA assays with Dunnet’s post-test using (**A**) intact supernatants, (**B**) LB++, (**C**) M9++, or (**D**) LB ++ as the control data (*P < 0.05. **P < 0.01, ***P < 0.001).

To better understand the molecule(s) produced by *B. cenocepacia*, supernatant from 7-day-old *B. cenocepacia* or *S. aureus* mono-culture biofilms was treated with enzymes that destroy DNA (DNaseI), RNA (RNaseA), or proteins (Proteinase K), boiled for 10 min, frozen for 2 h, or left “intact” with no treatment. These supernatants were then inoculated with *S. aureus* and incubated for 7 days to allow for biofilm production. As positive controls, *S. aureus* was also inoculated into medium alone or 1× PBS, and both of these media yielded relatively high concentrations of *S. aureus* after 7 days of incubation (Fig. 7A). *S. aureus* incubated with treated or untreated *S. aureus* biofilm supernatants supported biofilms with high levels of living *S. aureus* cells. In fact, *S. aureus* supernatants treated with DNaseI or DNaseI and Proteinase K together yielded significantly higher *S. aureus* concentrations than “intact” supernatants. In comparison, none of the enzymatic or temperature treatments of *B. cenocepacia* biofilm supernatants yielded detectable levels of *S. aureus* suggesting that DNA, RNA, and proteins are not the main inhibitory components within the *B. cenocepacia* supernatants.

We next tested whether certain polar metabolites could be responsible for *S. aureus* inhibition. To screen for polar metabolites produced by *B. cenocepacia* strains, 7-day biofilm supernatants from *S. aureus* NRS77, *B. cenocepacia* J2315, and H111 were filter sterilized, freeze dried, tested for *S. aureus* inhibition, and assessed through metabolomics. During sample preparation of biological replicates, one of the *B. cenocepacia* J2315 replicates did not inhibit *S. aureus*, while three others showed a strong inhibition. All replicate supernatants of *B. cenocepacia* H111 showed strong inhibition against *S. aureus*. Approximately 660 metabolites were identified in total from all conditions (supplemental file). We filtered the data set to identify metabolites that were (i) found at a substantially higher intensity in the *B. cenocepacia* J2315 biofilm supernatants with anti-*S*. *aureus* activity compared to the non-active *B. cenocepacia* J2315 biofilm supernatant, (ii) at least 10-fold more intensity in *B. cenocepacia* H111 and J2315 active supernatants compared to *S. aureus* biofilm supernatants and/or LB alone, and (iii) those with m/z ratios consistent with known bacterially produced metabolites. The latter was necessary as many metabolites that mapped to the databases had m/z ratios consistent with metabolites produced in plants or human cells. Therefore, for each candidate metabolite not known to be produced in bacteria (e.g., oryzaemate, sacchropine, hydroxyhexanoycarnitine), alternatives compounds with similar m/z ratios were found from primary literature studies and other databases and included in the list of top candidates. After filtering the data set, 13 candidate molecules were identified for further investigation (Table 2).

**TABLE 2:** Metabolites identified in this study filtered for inhibitory potential

Compound in database	m/z values^*a*^	Retention time^*a*^	J2315 non-active	J2315 active^*a*^	H111^*a*^	NRS77^*a*^	LB^*a*^	Bacterially produced alternative compounds with similar m/z ratios
Pyochelin	325.0671	18.15193	6.10E+05	4.16E+08	1.83E+09	3.88E+07	3.79E+07	
N-Acetylvaline	160.0968	5.458926	3.27E+07	1.55E+09	3.55E+09	1.31E+08	7.10E+07	
Oryzaemate	224.0375	17.22455	7.70E+06	1.56E+08	5.40E+08	6.74E+06	6.32E+06	5-aminonaphthalene-2-sulfonic acid
p-Methoxybenzyl-isothiocyanate	178.0335	17.22442	3.36E+06	5.32E+07	2.11E+08	8.04E+05	2.15E+06	
4,4-Bis[4-(acetyloxy)phenyl]3-hexanone	338.4044	1.141926	7.03E+06	1.13E+08	2.04E+08	1.96E+06	3.41E+06	
Isoxanthopterin	104.8623	2.971367	5.72E+05	1.39E+06	1.85E+06	2.50E−01	2.50E−01	
Saccharopine	252.1979	9.407097	3.01E+06	9.58E+06	2.07E+07	2.25E+05	4.89E+05	N-acetylglutamic acid
3-Methylglutarylcarnitine	288.1453	15.68265	2.09E+07	2.45E+07	6.57E+07	7.60E+05	1.34E+06	
g-Butyrobetaine	146.1175	5.185534	1.33E+07	4.56E+08	1.02E+09	2.74E+07	1.39E+07	
Uric acid	167.0214	5.528594	1.30E+08	2.74E+07	2.82E+08	4.40E+06	1.14E+06	
Urea	61.03977	4.454614	4.02E+08	3.69E+08	4.65E+08	1.52E+07	1.19E+07	
Gamma-glutamyl-gamma-glutamyl-N-carboxy-beta-alanine	228.5744	3.899862	7.51E+05	7.02E+05	1.30E+06	2.50E−01	2.50E−01	
Hydroxyhexanoycarnitine	274.1658	17.08677	1.33E+07	9.63E+06	2.68E+07	1.59E+05	6.25E+05	alpha-aminoadipic acid

^*a*^ Average values for all relevant replicates.

One of the top candidates that is known to be produced by *B. cenocepacia* was pyochelin, a siderophore. To test if pyochelin played a role in *S. aureus* inhibition, 3-day biofilm supernatants from a *B. cenocepacia* H111 strain bearing an in-frame deletion in the *pchAB* genes necessary for the production of pyochelin (35) were tested for inhibition of *S. aureus*. In parallel, supernatants from *B. cenocepacia* H111 with in-frame deletions of *orbJ*, a gene required for ornibactin production (another siderophore), and a double mutant unable to produce both ornibactin and pyochelin were also tested. As controls, wild-type *B. cenocepacia* H111 or *S. aureus* biofilm supernatants and LB medium alone were also tested. Medium alone and *S. aureus* supernatants allowed for robust *S. aureus* biofilm growth (Fig. 7B). As expected, supernatants from *B. cenocepacia* H111 wild type showed a completed inhibition of *S. aureus* biofilms. Supernatants from *B. cenocepacia* H111 Δ*orbJ* mutant were similar inhibitory to *S. aureus*. However, supernatants from *B. cenocepaci*a H111 Δ*pchAB* mutant and the H111 Δ*orbJ pchAB* double mutant yielded high *S. aureus* concentrations that were significantly different than the LB alone control but not statistically different from the *S. aureus* biofilm supernatant control. This suggests that pyochelin may underlie at least part of the inhibition observed.

To explore the role of iron availability further, two experiments were performed. First, exogenous iron (in the form of FeCl_3_) in a minimal medium on *S. aureus* in the absence of *B. cenocepacia* was explored. Low levels of iron did not support *S. aureus* biofilm formation, but 1 mM FeCl_3_•6H_2_O was sufficient to support *S. aureus* survival in biofilms (Fig. 7C).

In addition to pyochelin, other metabolites from the top candidates were tested for their ability to inhibit *S. aureus* biofilm formation. For these assays, supplemented LB was amended with differing concentrations of N-acetylvaline, urea, uric acid, N-acetylmuramic acid, aminoadipic acid, L-carnitine, or p-methoxybenzylisothiocyanate (4-MBITC) and used as the medium for *S. aureus* biofilm formation. After 3 days of incubation, only 4-MBITC showed a small but significant reduction in *S. aureus* survival. Therefore, other products potentially produced by *B. cenocepacia* may also contribute to the inhibitory effect against *S. aureus*.

## DISCUSSION

In this work, we describe a phenomenon in which a relatively slow-growing bacterium, *B. cenocepacia*, inhibits a much faster growing bacterium, *S. aureus*, during biofilm formation. This antagonism occurs both when these two bacteria are co-inoculated (i.e., prior to biofilm formation) and when *S. aureus* establishes a biofilm before addition of *B. cenocepacia* (Fig. 1). Further, we have shown that this phenomenon is mediated by a contact-free mechanism, which leads to *S. aureus* cell death, and this mechanism is not due to *B. cenocepacia* simply acidifying the culture environment (Fig. 4 to 6; Fig. S3). This interaction also is unlikely to be due to competition for resources as *S. aureus* can grow at a much faster rate in planktonic cultures than *B. cenocepacia* under these conditions, but the nutritional/metabolic status may be important for *S. aureus* sensitivity to these compound(s) as this inhibition was not observed in all growth media (Fig. S4).

Because the expression of the inhibitory substance seems to be secreted during biofilm formation in *B. cenocepacia*, one might ask whether quorum sensing plays a role in the regulation of the inhibitory substance(s). As the CepI/CepR quorum-sensing systems tend to increase in acyl-homoserine lactone (AHL) production up 24 h and is known to be involved in biofilm formation (38), it is entirely possible that the accumulation of AHL triggers the expression of the inhibitory substance in *B. cenocepacia* J2315 biofilms. However, we note that overnight cultures of *B. cenocepacia* were unable to inhibit *S. aureus* (Fig. 4), so the simple model of high cell density alone cannot explain the biosynthesis of the inhibitory substance. Further, at least one group has observed that *B. multivorans* does not secrete AHLs despite the presence of quorum-sensing genes in the genome, and yet, *B. multivorans* still inhibited *S. aureus* when in co-culture (Fig. 3). Thus, whether quorum sensing contributes to the biosynthesis/regulation of the inhibitory substance(s) that targets *S. aureus* is an open area of interest.

Other antimicrobial compounds produced by *Burkholderia* species have been explored as the large plastic genomes are prone to gene acquisition. Species within *Burkholderia* have been shown to produce nitrogen-containing volatile organic compounds (such as pyrrolnitrin, phenazines, and indoles), other volatile organic compounds (such as methyl anthranilate, dimethyl disulfide, and nonanoic acid, polyenes, siderophores like cepabactin and ornibactin, bacteriocins), and other compounds [reviewed in (39)]. However, these compounds have either not been described for *Burkholderia* species in the Bcc or they are narrow-range antimicrobials that target closely related species or distantly related species like fungi.

*P. aeruginosa*, a bacterial pathogen known to often interact with *S. aureus* in the lungs of people with CF, has evolved at least three mechanisms to inhibit *S. aureus*. First, secondary metabolites, including 4-hydroxy-2-heptylquinoline-N-oxide (HQNO), pyocyanin, and hydrogen cyanide, function to disrupt *S. aureus* growth by inhibiting aerobic respiration (40–43). Additionally, *P. aeruginosa* produces a mixture of rhamnolipids that loosens the membrane of *S. aureus* cells and dislodges *S. aureus* from biofilms (44–46) and makes them more susceptible to antimicrobial agents (47). Finally, *P. aeruginosa* makes an endopeptidase, LasA, that acts much like lysostaphin (48), and this protein, like rhamnolipids, can enhance the efficacy of antibiotics on *S. aureus* (43). Interestingly, *B. cenocepacia* J2315 lacks appreciable homologs to the LasA and Pqs proteins (for HQNO production) in its genome but has *rhlABC* homologs responsible for making rhamnolipids on its second chromosome (49). However, there are no published reports that describe rhamnolipid production in *B. cenocepacia* species. In the related species *Burkholderia thailandensis* and in *B. pseudomallei*, homologs of these genes are associated with the production of rhamnolipids (50). Our data suggest that a BSL-2-strain of *B. pseudomallei* can inhibit *S. aureus* almost as well as *B. cenocepacia* strains (Fig. 3C).

In this study, proteinase K treatment of *B. cenocepacia* biofilm supernatants did not reduce the inhibition of *S. aureus* suggesting that a protein-mediated inhibition is unlikely. Proteinase K is a broad-spectrum serine protease that can cleave most, but not all, proteins (51); thus, a proteinase K-resistant protein could still serve as a potential mediator of *S. aureus* inhibition. Additionally, a wide range of polar metabolites are produced by *B. cenocepacia* during biofilm formation, and a few of these were shown in this study to contribute to *B. cenocepacia* inhibition of *S. aureus*. Notably, pyochelin, a siderophore, seems to contribute most/all of the inhibition (Fig. 7). Pyochelin is one of several siderophores produced by *B. cenocepacia* isolates (52–55) and has been implicated in virulence in lung infections in animal models but is not essential for plant or invertebrate organisms (35, 56, 57). Additionally, *B. pseudomallei* has also been shown to produce pyochelin (58) and thus could explain the *B. pseudomalle*i inhibition of *S. aureus* shown in this study (Fig. 3). Pyochelin has been shown to have anti-staphylococcal properties (59, 60). Further Uzi-Gavrilov et al. recently showed that *S. aureus* converts *P. aeruginosa* pyochelin to a pyochelin methyl ester, which then acts as a signal to change gene expression in *P. aeruginosa*, thus acting as a signaling molecule (61) and additional studies have shown that the toxic effects of pyochelin on other bacteria may be mediated through the production of reactive oxygen species (59, 60). Notably, a recent study showed that some strains of *S. aureus* produce a methylase that detoxifies the toxic effects of pyochelin during planktonic growth (62). In this study, the *S. aureus* strain used (NRS77; aka NCTC 8325) encodes the methylase described in this study (annotated as a class I SAM-dependent methyltransferase) but it is either not expressed in this medium, under biofilm conditions, or it is not able to methylate the pyochelin of *B. cenocepacia* specifically. Another recent study showed that *Burkholderia paludis*, a more distantly related *Burkholderia* strain, produces a pyochelin capable of inhibiting Gram-positive species, including *S. aureus*, through generation of reactive oxygen species within the cytoplasm of the target cell (63). Whether the *B. cenocepacia* pyochelin-mediated inhibition of *S. aureus* during biofilm growth and maintenance is via a signaling system, iron sequestration, reactive oxygen species, or another mechanism during biofilm formation remains to be determined.

**Fig 8:**
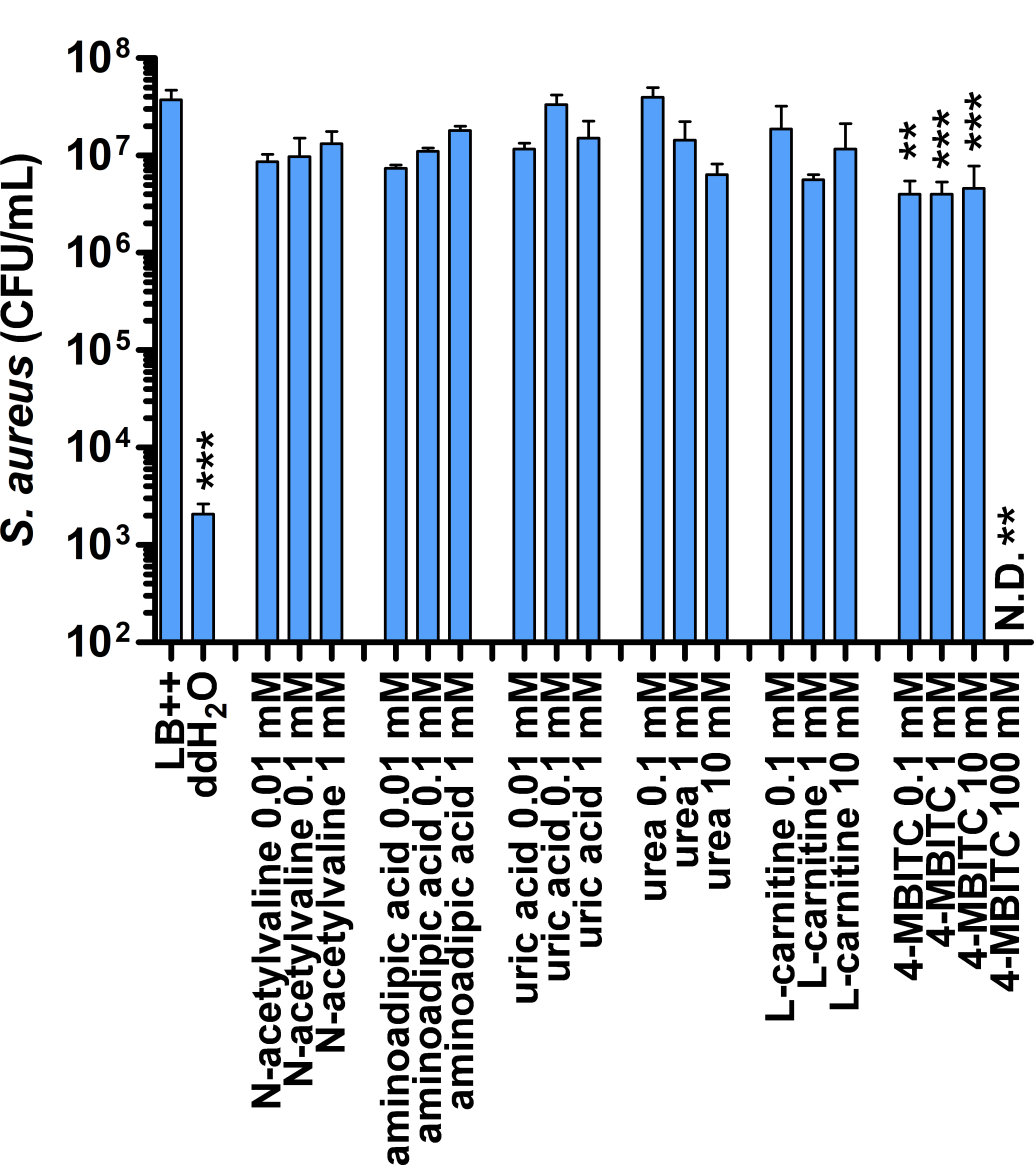
The effect of polar metabolites on *B. cenocepacia* inhibition of *S. aureus*. Purified metabolites were added to LB++ medium as indicated. *S. aureus* survival after 3 days of biofilm formation in these media was quantified. Statistically significant changes based on one-way ANOVAs with Dunnet’s post-test using LB++ as a control are indicated with asterisks **P < 0.01, ***P < 0.001).

Other metabolites may also play a role in *S. aureus* inhibition. 4-MBITC showed a mild inhibition (approximately eightfold) when added exogenously to *S. aureus* (Fig. 8). This compound, produced by plant species, is part of a broader class of compounds, known as glucosinolates, that can be converted into isothiocyanates by microbes, particularly those in the human gut (64), and these converted isothiocyanates, including 4-MBITC, have been shown to have antimicrobial activities (65). While *B. cenocepacia* is not known to produce glucosinolates itself, the medium used contains plant extracts that could potentially be a source of these molecules, or this pathway in *B. cenocepacia* could be yet undefined. Future directions will include testing additional metabolites and examining the roles of lipids in the *B. cenocepacia*-mediated inhibition of *S. aureus*.

In summary, *S. aureus* is a bacterial pathogen of significant importance, especially in clinical settings where it can cause a wide range of infections, many of which are life threatening. Elucidation of the mechanism and identification of secreted products from individual bacterial species (alone or in combination with those produced by multiple species) that can target and disrupt either the survival or biofilm formation or maintenance of *S. aureus* may lead to new therapeutic targets or treatments that improve efficacy of currently available antibiotics or set the stage for new narrow-range antimicrobials. This work identifies at least two potential new mechanisms that might be useful for combatting this extremely common, and sometimes deadly, bacterial pathogen.

## Data Availability

The raw data have been uploaded to Figshare and can be accessed here: https://doi.org/10.6084/m9.figshare.28543700.v1.

## SUPPLEMENTAL MATERIAL

Figure S1 jb.00116-23-s0001.tif Optimization co-culture biofilm growth.

Figure S2 jb.00116-23-s0002.tif Effect of pH and pigments on inhibition of *S. aureus*.

Figure S3 jb.00116-23-s0003.tif Supernatants from other Burks effect on *S. aureus*.

Figure S4 jb.00116-23-s0004.tif Effect of medium types on inhibition.

Figure S5 jb.00116-23-s0005.tif Biofilm vs planktonic *S. aureus* GFP and propidium iodide quantitation.

Figure S6 jb.00116-23-s0006.tif Confocal images.

Supplemental File jb.00116-23-s0007.xlsx All metabolomics data for *B. cenocepacia*, *S. aureus* biofilm supernatants and LB++ medium.

ASM does not own the copyrights to Supplemental Material that may be linked to, or accessed through, an article. The authors have granted ASM a non-exclusive, world-wide license to publish the Supplemental Material files. Please contact the corresponding author directly for reuse.

## References

[1] Gordon RJ, Lowy FD. 2008. Pathogenesis of methicillin-resistant Staphylococcus aureus infection. Clin Infect Dis 46 Suppl 5:S350–S359. doi:10.1086/533591 18462090 PMC2474459

[2] Keynan Y, Rubinstein E. 2013. Staphylococcus aureus bacteremia, risk factors, complications, and management. Crit Care Clin 29:547–562. doi:10.1016/j.ccc.2013.03.008 23830653

[3] Keynan Y, Rubinstein E. 2013. Pathophysiology of infective endocarditis. Curr Infect Dis Rep 15:342–346. doi:10.1007/s11908-013-0346-0 23737237

[4] Le TT, Nadimpalli M, Wu J, Heaney CD, Stewart JR. 2018. Challenges in estimating characteristics of Staphylococcus aureus nasal carriage among humans enrolled in surveillance studies. Front Public Health 6:163. doi:10.3389/fpubh.2018.00163 29911098 PMC5992268

[5] Frank DN, Feazel LM, Bessesen MT, Price CS, Janoff EN, Pace NR. 2010. The human nasal microbiota and Staphylococcus aureus carriage. PLoS One 5:e10598. doi:10.1371/journal.pone.0010598 20498722 PMC2871794

[6] Otto M. 2010. Staphylococcus colonization of the skin and antimicrobial peptides. Expert Rev Dermatol 5:183–195. doi:10.1586/edm.10.6 20473345 PMC2867359

[7] Conlon BP. 2014. Staphylococcus aureus chronic and relapsing infections: evidence of a role for persister cells: an investigation of persister cells, their formation and their role in S. aureus disease. Bioessays 36:991–996. doi:10.1002/bies.201400080 25100240

[8] Wan N, Wang H, Ng CK, Mukherjee M, Ren D, Cao B, Tang YJ. 2018. Bacterial metabolism during biofilm growth investigated by ^13^C tracing. Front Microbiol 9:2657. doi:10.3389/fmicb.2018.02657 30515135 PMC6255981

[9] Foster TJ. 2017. Antibiotic resistance in Staphylococcus aureus. Current status and future prospects. FEMS Microbiol Rev 41:430–449. doi:10.1093/femsre/fux007 28419231

[10] Sweeney E, Harrington NE, Harley Henriques AG, Hassan MM, Crealock-Ashurst B, Smyth AR, Hurley MN, Tormo-Mas MÁ, Harrison F. 2021. An ex vivo cystic fibrosis model recapitulates key clinical aspects of chronic Staphylococcus aureus infection. Microbiol (Reading, Engl) 167. doi:10.1099/mic.0.000987 33186093

[11] Jean-Pierre V, Boudet A, Sorlin P, Menetrey Q, Chiron R, Lavigne JP, Marchandin H. 2022. Biofilm formation by Staphylococcus aureus in the specific context of cystic fibrosis. Int J Mol Sci 24:597. doi:10.3390/ijms24010597 36614040 PMC9820612

[12] Foundation CF. 2021. Cystic fibrosis foundation annual report 2021. Cystic Fibrosis Foundation, Bethesda, MD.

[13] Govan JR, Deretic V. 1996. Microbial pathogenesis in cystic fibrosis: mucoid Pseudomonas aeruginosa and Burkholderia cepacia. Microbiol Rev 60:539–574. doi:10.1128/mr.60.3.539-574.1996 8840786 PMC239456

[14] Speert DP, Henry D, Vandamme P, Corey M, Mahenthiralingam E. 2002. Epidemiology of Burkholderia cepacia complex in patients with cystic fibrosis, Canada. Emerg Infect Dis 8:181–187. doi:10.3201/eid0802.010163 11897071 PMC3369581

[15] Torbeck L, Raccasi D, Guilfoyle DE, Friedman RL, Hussong D. 2011. Burkholderia cepacia: this decision is overdue. PDA J Pharm Sci Technol 65:535–543. doi:10.5731/pdajpst.2011.00793 22293841

[16] Cuthbertson L, Walker AW, Oliver AE, Rogers GB, Rivett DW, Hampton TH, Ashare A, Elborn JS, De Soyza A, Carroll MP, Hoffman LR, Lanyon C, Moskowitz SM, O’Toole GA, Parkhill J, Planet PJ, Teneback CC, Tunney MM, Zuckerman JB, Bruce KD, van der Gast CJ. 2020. Lung function and microbiota diversity in cystic fibrosis. Microbiome 8:45. doi:10.1186/s40168-020-00810-3 32238195 PMC7114784

[17] Flight WG, Smith A, Paisey C, Marchesi JR, Bull MJ, Norville PJ, Mutton KJ, Webb AK, Bright-Thomas RJ, Jones AM, Mahenthiralingam E. 2015. Rapid detection of emerging pathogens and loss of microbial diversity associated with severe lung disease in cystic fibrosis. J Clin Microbiol 53:2022–2029. doi:10.1128/JCM.00432-15 25878338 PMC4473198

[18] Zemanick ET, Wagner BD, Robertson CE, Ahrens RC, Chmiel JF, Clancy JP, Gibson RL, Harris WT, Kurland G, Laguna TA, McColley SA, McCoy K, Retsch-Bogart G, Sobush KT, Zeitlin PL, Stevens MJ, Accurso FJ, Sagel SD, Harris JK. 2017. Airway microbiota across age and disease spectrum in cystic fibrosis. Eur Respir J 50:1700832. doi:10.1183/13993003.00832-2017 29146601 PMC5935257

[19] Zlosnik JEA, Zhou G, Brant R, Henry DA, Hird TJ, Mahenthiralingam E, Chilvers MA, Wilcox P, Speert DP. 2015. Burkholderia species infections in patients with cystic fibrosis in British Columbia, Canada. 30 years’ experience. Ann Am Thorac Soc 12:70–78. doi:10.1513/AnnalsATS.201408-395OC 25474359

[20] Veech JA. 2013. A probabilistic model for analysing species co-occurrence. Glob Ecol Biogeogr 22:252–260. doi:10.1111/j.1466-8238.2012.00789.x

[21] Griffith DM, Veech JA, Marsh CJ. 2016. cooccur: probabilistic species co-occurrence analysis in R. J Stat Softw 69:1–17. doi:10.18637/jss.v069.c02

[22] Pang YY, Schwartz J, Thoendel M, Ackermann LW, Horswill AR, Nauseef WM. 2010. agr-dependent interactions of Staphylococcus aureus USA300 with human polymorphonuclear neutrophils. J Innate Immun 2:546–559. doi:10.1159/000319855 20829608 PMC2982852

[23] Holden MTG, Seth-Smith HMB, Crossman LC, Sebaihia M, Bentley SD, Cerdeño-Tárraga AM, Thomson NR, Bason N, Quail MA, Sharp S, et al.. 2009. The genome of Burkholderia cenocepacia J2315, an epidemic pathogen of cystic fibrosis patients. J Bacteriol 191:261–277. doi:10.1128/JB.01230-08 18931103 PMC2612433

[24] Morales-Ruíz L-M, Rodríguez-Cisneros M, Kerber-Díaz J-C, Rojas-Rojas F-U, Ibarra JA, Estrada-de los Santos P. 2022. Burkholderia orbicola sp. nov., a novel species within the Burkholderia cepacia complex. Arch Microbiol 204. doi:10.1007/s00203-022-02778-0 35174425

[25] Gonyar LA, Fankhauser SC, Goldberg JB. 2015. Single amino acid substitution in homogentisate 1,2-dioxygenase is responsible for pigmentation in a subset of Burkholderia cepacia complex isolates. Environ Microbiol Rep 7:180–187. doi:10.1111/1758-2229.12217 25294803 PMC4560265

[26] LiPuma JJ, Spilker T, Coenye T, Gonzalez CF. 2002. An epidemic Burkholderia cepacia complex strain identified in soil. Lancet 359:2002–2003. doi:10.1016/S0140-6736(02)08836-0 12076559

[27] Carlier A, Agnoli K, Pessi G, Suppiger A, Jenul C, Schmid N, Tümmler B, Pinto-Carbo M, Eberl L. 2014. Genome sequence of Burkholderia cenocepacia H111, a cystic fibrosis airway isolate. Genome Announc 2:e00298-14. doi:10.1128/genomeA.00298-14 24723723 PMC3983312

[28] Baldwin A, Mahenthiralingam E, Thickett KM, Honeybourne D, Maiden MCJ, Govan JR, Speert DP, Lipuma JJ, Vandamme P, Dowson CG. 2005. Multilocus sequence typing scheme that provides both species and strain differentiation for the Burkholderia cepacia complex. J Clin Microbiol 43:4665–4673. doi:10.1128/JCM.43.9.4665-4673.2005 16145124 PMC1234123

[29] Keith KE, Killip L, He P, Moran GR, Valvano MA. 2007. Burkholderia cenocepacia C5424 produces a pigment with antioxidant properties using a homogentisate intermediate. J Bacteriol 189:9057–9065. doi:10.1128/JB.00436-07 17933889 PMC2168628

[30] Chen JS, Witzmann KA, Spilker T, Fink RJ, LiPuma JJ. 2001. Endemicity and inter-city spread of Burkholderia cepacia genomovar III in cystic fibrosis. J Pediatr 139:643–649. doi:10.1067/mpd.2001.118430 11713440

[31] Jacobs JL, Fasi AC, Ramette A, Smith JJ, Hammerschmidt R, Sundin GW. 2008. Identification and onion pathogenicity of Burkholderia cepacia complex isolates from the onion rhizosphere and onion field soil. Appl Environ Microbiol 74:3121–3129. doi:10.1128/AEM.01941-07 18344334 PMC2394932

[32] Vermis K, Coenye T, LiPuma JJ, Mahenthiralingam E, Nelis HJ, Vandamme P. 2004. Proposal to accommodate Burkholderia cepacia genomovar VI as Burkholderia dolosa sp. nov. Int J Syst Evol Microbiol 54:689–691. doi:10.1099/ijs.0.02888-0 15143009

[33] Varga JJ, Losada L, Zelazny AM, Brinkac L, Harkins D, Radune D, Hostetler J, Sampaio EP, Ronning CM, Nierman WC, Greenberg DE, Holland SM, Goldberg JB. 2012. Draft genome sequence determination for cystic fibrosis and chronic granulomatous disease Burkholderia multivorans isolates. J Bacteriol 194:6356–6357. doi:10.1128/JB.01306-12 23105085 PMC3486389

[34] Propst KL, Mima T, Choi KH, Dow SW, Schweizer HP. 2010. A Burkholderia pseudomallei deltapurM mutant is avirulent in immunocompetent and immunodeficient animals: candidate strain for exclusion from select-agent lists. Infect Immun 78:3136–3143. doi:10.1128/IAI.01313-09 20404077 PMC2897367

[35] Mathew A, Eberl L, Carlier AL. 2014. A novel siderophore-independent strategy of iron uptake in the genus Burkholderia. Mol Microbiol 91:805–820. doi:10.1111/mmi.12499 24354890

[36] Zhao J, Schloss PD, Kalikin LM, Carmody LA, Foster BK, Petrosino JF, Cavalcoli JD, VanDevanter DR, Murray S, Li JZ, Young VB, LiPuma JJ. 2012. Decade-long bacterial community dynamics in cystic fibrosis airways. Proc Natl Acad Sci U S A 109:5809–5814. doi:10.1073/pnas.1120577109 22451929 PMC3326496

[37] Palmer KL, Aye LM, Whiteley M. 2007. Nutritional cues control Pseudomonas aeruginosa multicellular behavior in cystic fibrosis sputum. J Bacteriol 189:8079–8087. doi:10.1128/JB.01138-07 17873029 PMC2168676

[38] Schmid N, Pessi G, Deng Y, Aguilar C, Carlier AL, Grunau A, Omasits U, Zhang LH, Ahrens CH, Eberl L. 2012. The AHL- and BDSF-dependent quorum sensing systems control specific and overlapping sets of genes in Burkholderia cenocepacia H111. PLoS One 7:e49966. doi:10.1371/journal.pone.0049966 23185499 PMC3502180

[39] Rodríguez-Cisneros M, Morales-Ruíz LM, Salazar-Gómez A, Rojas-Rojas FU, Estrada-de Los Santos P. 2023. Compilation of the antimicrobial compounds produced by Burkholderia sensu stricto. Molecules 28:1646. doi:10.3390/molecules28041646 36838633 PMC9958762

[40] Filkins LM, Graber JA, Olson DG, Dolben EL, Lynd LR, Bhuju S, O’Toole GA. 2015. Coculture of Staphylococcus aureus with Pseudomonas aeruginosa drives S. aureus towards fermentative metabolism and reduced viability in a cystic fibrosis model. J Bacteriol 197:2252–2264. doi:10.1128/JB.00059-15 25917910 PMC4524177

[41] Caldelari I, Chao Y, Romby P, Vogel J. 2013. RNA-mediated regulation in pathogenic bacteria. Cold Spring Harb Perspect Med 3:a010298. doi:10.1101/cshperspect.a010298 24003243 PMC3753719

[42] Orazi G, Ruoff KL, O’Toole GA. 2019. Pseudomonas aeruginosa increases the sensitivity of biofilm-grown Staphylococcus aureus to membrane-targeting antiseptics and antibiotics. MBio 10:e01501-19. doi:10.1128/mBio.01501-19 31363032 PMC6667622

[43] Radlinski L, Rowe SE, Kartchner LB, Maile R, Cairns BA, Vitko NP, Gode CJ, Lachiewicz AM, Wolfgang MC, Conlon BP. 2017. Pseudomonas aeruginosa exoproducts determine antibiotic efficacy against Staphylococcus aureus. PLoS Biol 15:e2003981. doi:10.1371/journal.pbio.2003981 29176757 PMC5720819

[44] Nguyen AT, Jones JW, Cámara M, Williams P, Kane MA, Oglesby-Sherrouse AG. 2016. Cystic fibrosis isolates of Pseudomonas aeruginosa retain iron-regulated antimicrobial activity against Staphylococcus aureus through the action of multiple alkylquinolones. Front Microbiol 7:1171. doi:10.3389/fmicb.2016.01171 27512392 PMC4961689

[45] Bharali P, Saikia JP, Ray A, Konwar BK. 2013. Rhamnolipid (RL) from Pseudomonas aeruginosa OBP1: a novel chemotaxis and antibacterial agent. Colloids Surf B Bio 103:502–509. doi:10.1016/j.colsurfb.2012.10.064 23261573

[46] Soberón-Chávez G, Lépine F, Déziel E. 2005. Production of rhamnolipids by Pseudomonas aeruginosa. Appl Microbiol Biotechnol 68:718–725. doi:10.1007/s00253-005-0150-3 16160828

[47] Wood TL, Gong T, Zhu L, Miller J, Miller DS, Yin B, Wood TK. 2018. Rhamnolipids from Pseudomonas aeruginosa disperse the biofilms of sulfate-reducing bacteria. NPJ Biofilms Microbiomes 4:22. doi:10.1038/s41522-018-0066-1 30302271 PMC6170446

[48] Kessler E, Safrin M, Olson JC, Ohman DE. 1993. Secreted LasA of Pseudomonas aeruginosa is a staphylolytic protease. J Biol Chem 268:7503–7508. doi:10.1016/S0021-9258(18)53203-8 8463280

[49] Fang K, Zhao H, Sun C, Lam CMC, Chang S, Zhang K, Panda G, Godinho M, Martins dos Santos VAP, Wang J. 2011. Exploring the metabolic network of the epidemic pathogen Burkholderia cenocepacia J2315 via genome-scale reconstruction. BMC Syst Biol 5:83. doi:10.1186/1752-0509-5-83 21609491 PMC3123600

[50] Dubeau D, Déziel E, Woods DE, Lépine F. 2009. Burkholderia thailandensis harbors two identical rhl gene clusters responsible for the biosynthesis of rhamnolipids. BMC Microbiol 9:263. doi:10.1186/1471-2180-9-263 20017946 PMC2804600

[51] Butler GH, Kotani H, Kong L, Frick M, Evancho S, Stanbridge EJ, McGarrity GJ. 1991. Identification and characterization of proteinase K-resistant proteins in members of the class Mollicutes. Infect Immun 59:1037–1042. doi:10.1128/iai.59.3.1037-1042.1991 1997407 PMC258364

[52] Darling P, Chan M, Cox AD, Sokol PA. 1998. Siderophore production by cystic fibrosis isolates of Burkholderia cepacia. Infect Immun 66:874–877. doi:10.1128/IAI.66.2.874-877.1998 9453660 PMC107988

[53] Sokol PA. 1986. Production and utilization of pyochelin by clinical isolates of Pseudomonas cepacia. J Clin Microbiol 23:560–562. doi:10.1128/jcm.23.3.560-562.1986 2937804 PMC268694

[54] Thomas MS. 2007. Iron acquisition mechanisms of the Burkholderia cepacia complex. Biometals 20:431–452. doi:10.1007/s10534-006-9065-4 17295049

[55] Tyrrell J, Whelan N, Wright C, Sá-Correia I, McClean S, Thomas M, Callaghan M. 2015. Investigation of the multifaceted iron acquisition strategies of Burkholderia cenocepacia. Biometals 28:367–380. doi:10.1007/s10534-015-9840-1 25725797

[56] Visser MB, Majumdar S, Hani E, Sokol PA. 2004. Importance of the ornibactin and pyochelin siderophore transport systems in Burkholderia cenocepacia lung infections. Infect Immun 72:2850–2857. doi:10.1128/IAI.72.5.2850-2857.2004 15102796 PMC387874

[57] Sokol PA, Woods DE. 1988. Effect of pyochelin on Pseudomonas cepacia respiratory infections. Microb Pathog 5:197–205. doi:10.1016/0882-4010(88)90022-8 3216778

[58] Alice AF, López CS, Lowe CA, Ledesma MA, Crosa JH. 2006. Genetic and transcriptional analysis of the siderophore malleobactin biosynthesis and transport genes in the human pathogen Burkholderia pseudomallei K96243. J Bacteriol 188:1551–1566. doi:10.1128/JB.188.4.1551-1566.2006 16452439 PMC1367220

[59] Adler C, Corbalán NS, Seyedsayamdost MR, Pomares MF, de Cristóbal RE, Clardy J, Kolter R, Vincent PA. 2012. Catecholate siderophores protect bacteria from pyochelin toxicity. PLoS ONE 7:e46754. doi:10.1371/journal.pone.0046754 23071628 PMC3465284

[60] Marques-Carvalho A, Kim H-N, Almeida M. 2023. The role of reactive oxygen species in bone cell physiology and pathophysiology. Bone Rep 19:101664. doi:10.1016/j.bonr.2023.101664 38163012 PMC10757300

[61] Uzi‐Gavrilov S, Tik Z, Sabti O, Meijler MM. 2023. Chemical modification of a bacterial siderophore by a competitor in dual-species biofilms. Angew Chem Int Ed 62. doi:10.1002/anie.202300585 37211536

[62] Jenul C, Keim KC, Jens JN, Zeiler MJ, Schilcher K, Schurr MJ, Melander C, Phelan VV, Horswill AR. 2023. Pyochelin biotransformation by Staphylococcus aureus shapes bacterial competition with Pseudomonas aeruginosa in polymicrobial infections. Cell Rep 42:112540. doi:10.1016/j.celrep.2023.112540 37227819 PMC10592502

[63] Ong KS, Cheow YL, Lee SM. 2017. The role of reactive oxygen species in the antimicrobial activity of pyochelin. J Adv Res 8:393–398. doi:10.1016/j.jare.2017.05.007 28580180 PMC5447373

[64] Sikorska-Zimny K, Beneduce L. 2021. The metabolism of glucosinolates by gut microbiota. Nutrients 13:2750. doi:10.3390/nu13082750 34444909 PMC8401010

[65] Iwu MW, Unaeze NC, Okunji CO, Corley DG, Sanson DR, Tempesta MS. 1991. Antibacterial aromatic isothiocyanates from the essential oil of Hippocratea welwitschii roots. Int J Pharmacogn 29:154–158. doi:10.3109/13880209109082869

