# Supplementary figures and images for: Burkholderia cenocepacia-mediated inhibition of Staphylococcus aureus growth and biofilm formation
Source: J Bacteriol. 2025 Mar 27;207(4):e00116-23. doi: 10.1128/jb.00116-23 (PMC12004965; doi:10.1128/jb.00116-23)

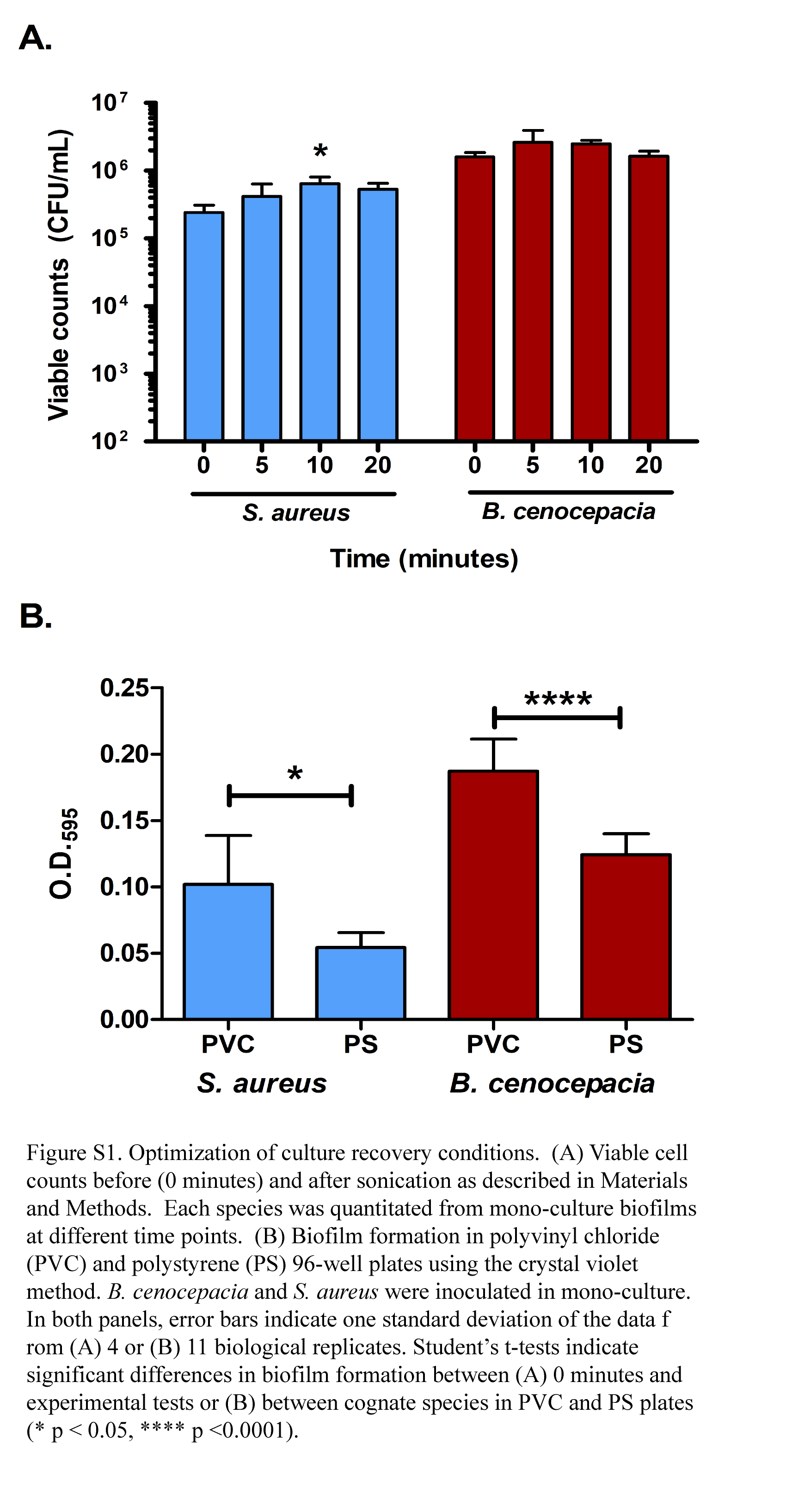

Supplement: Figure S1 — Optimization co-culture biofilm growth. [file jb.00116-23-s0001.tif]

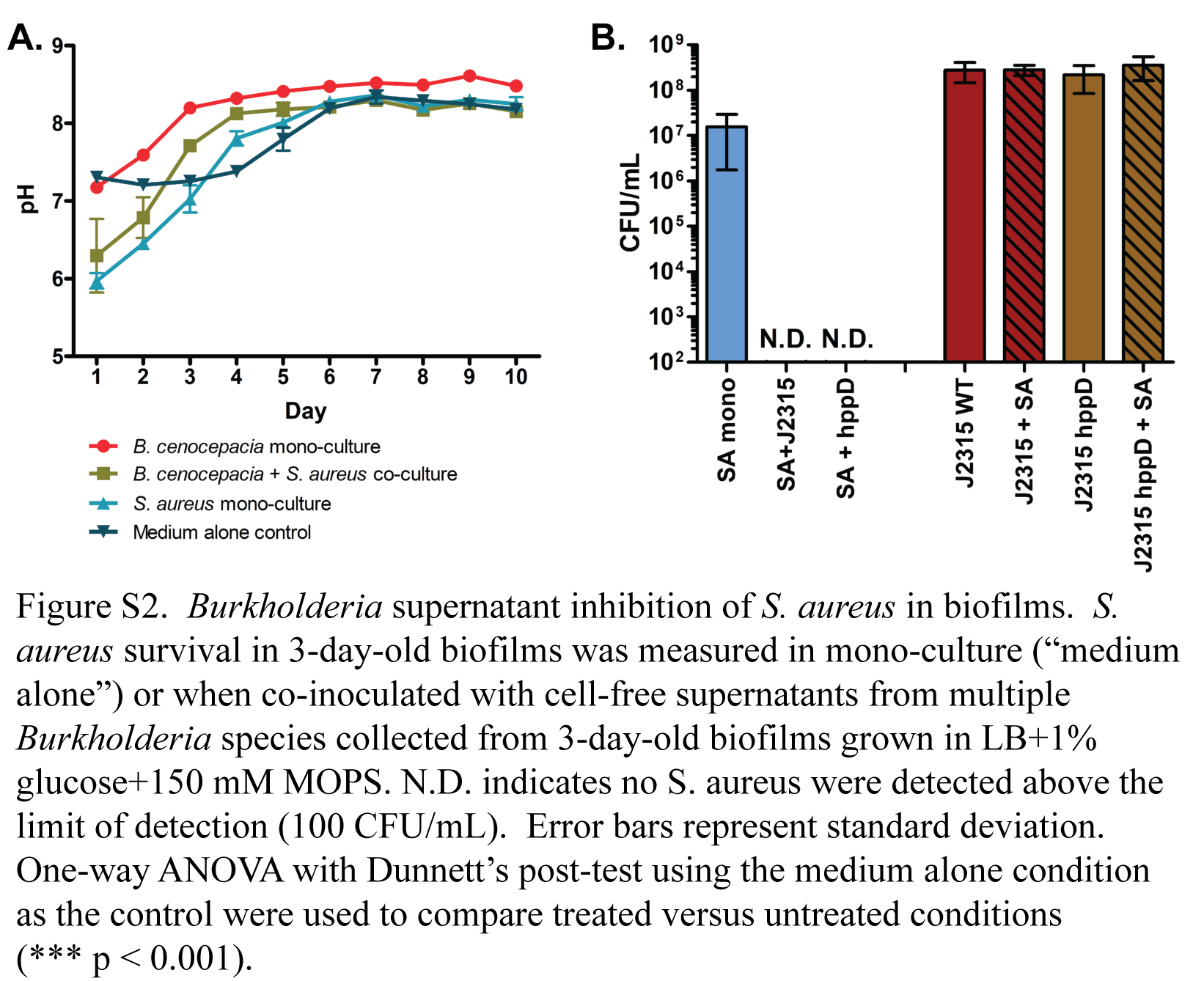

Supplement: Figure S2 — Effect of pH and pigments on inhibition of S. aureus. [file jb.00116-23-s0002.tif]

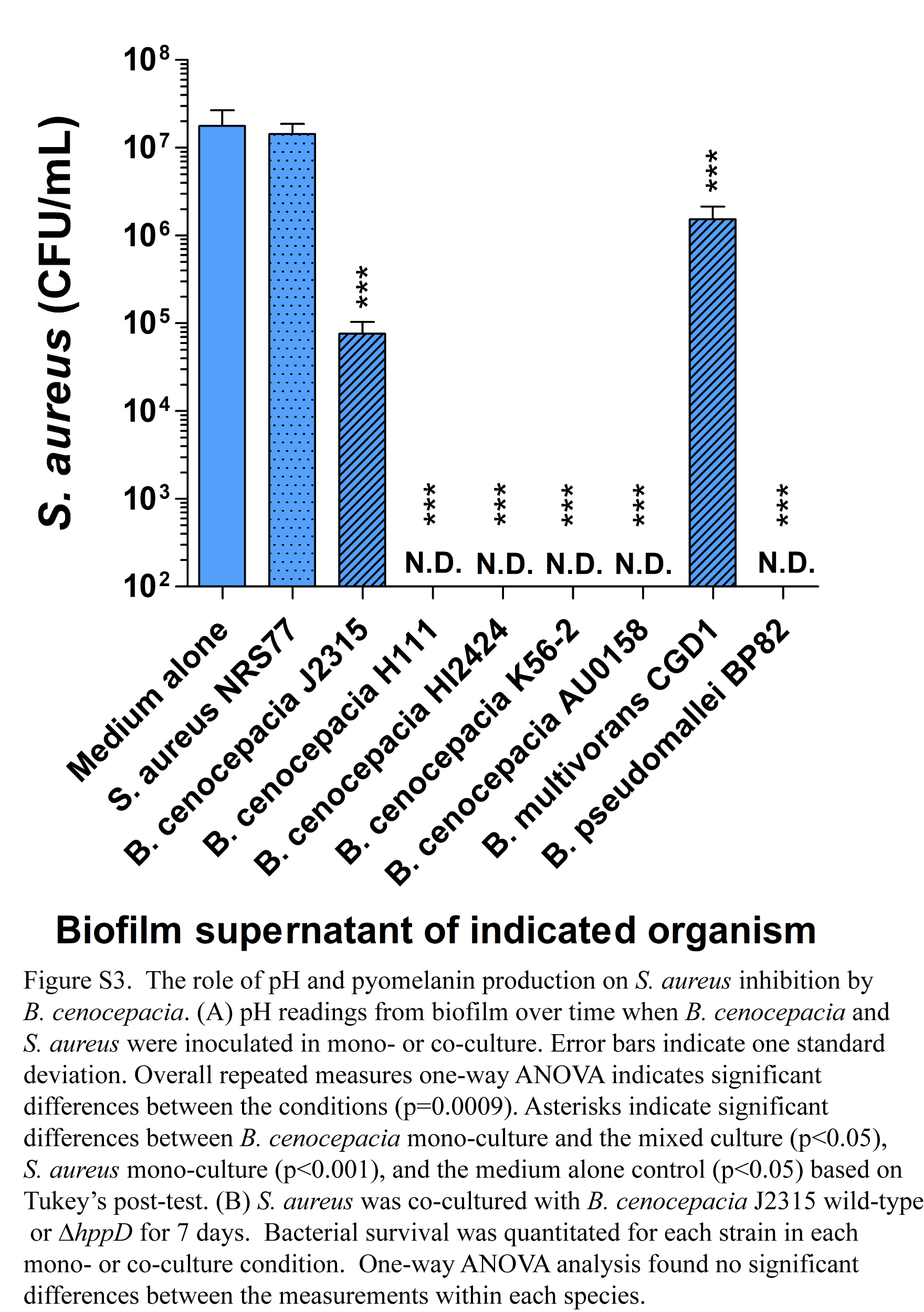

Supplement: Figure S3 — Supernatants from other Burks effect on S. aureus. [file jb.00116-23-s0003.tif]

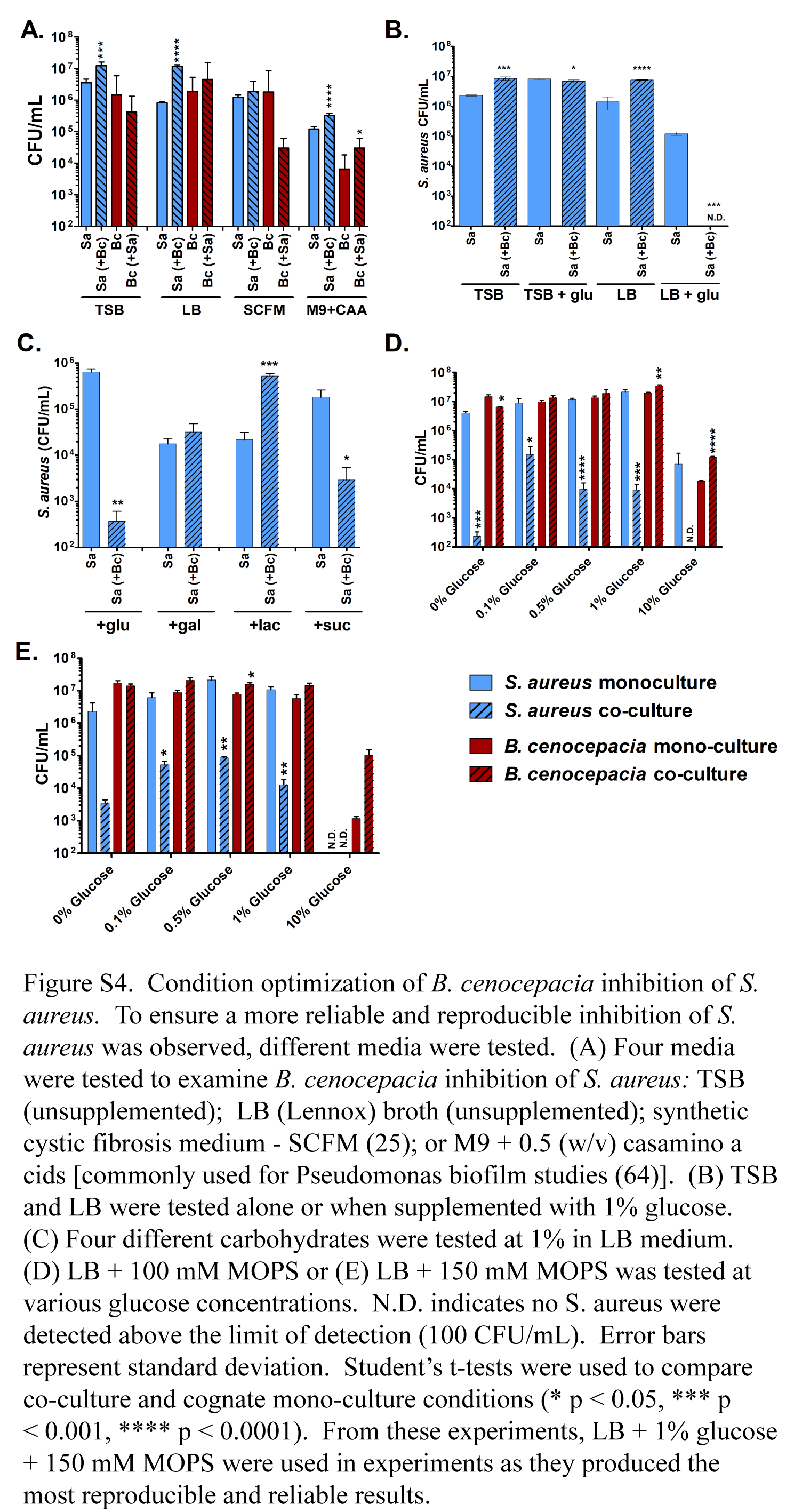

Supplement: Figure S4 — Effect of medium types on inhibition. [file jb.00116-23-s0004.tif]

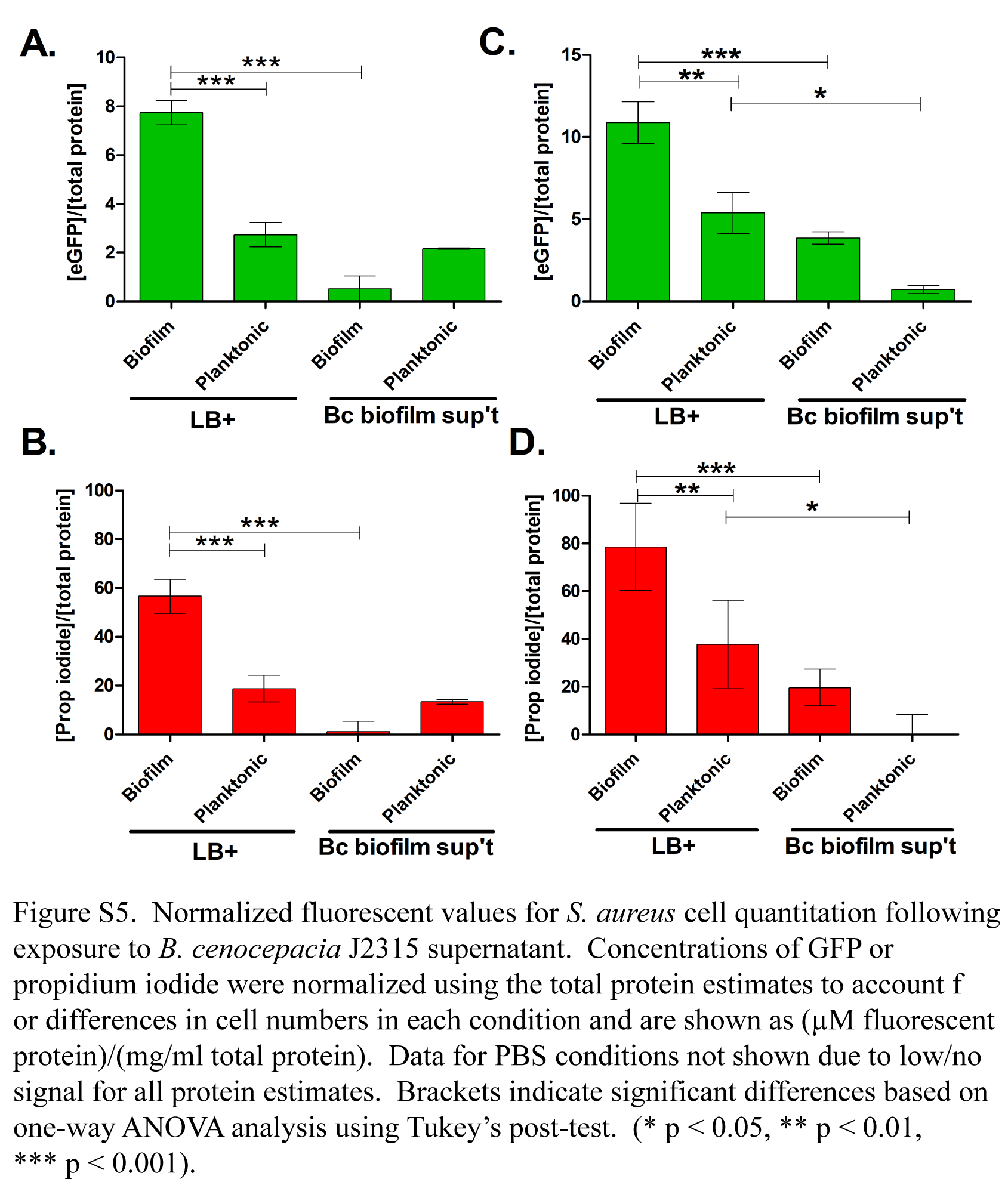

Supplement: Figure S5 — Biofilm vs planktonic S. aureus GFP and propidium iodide quantitation. [file jb.00116-23-s0005.tif]

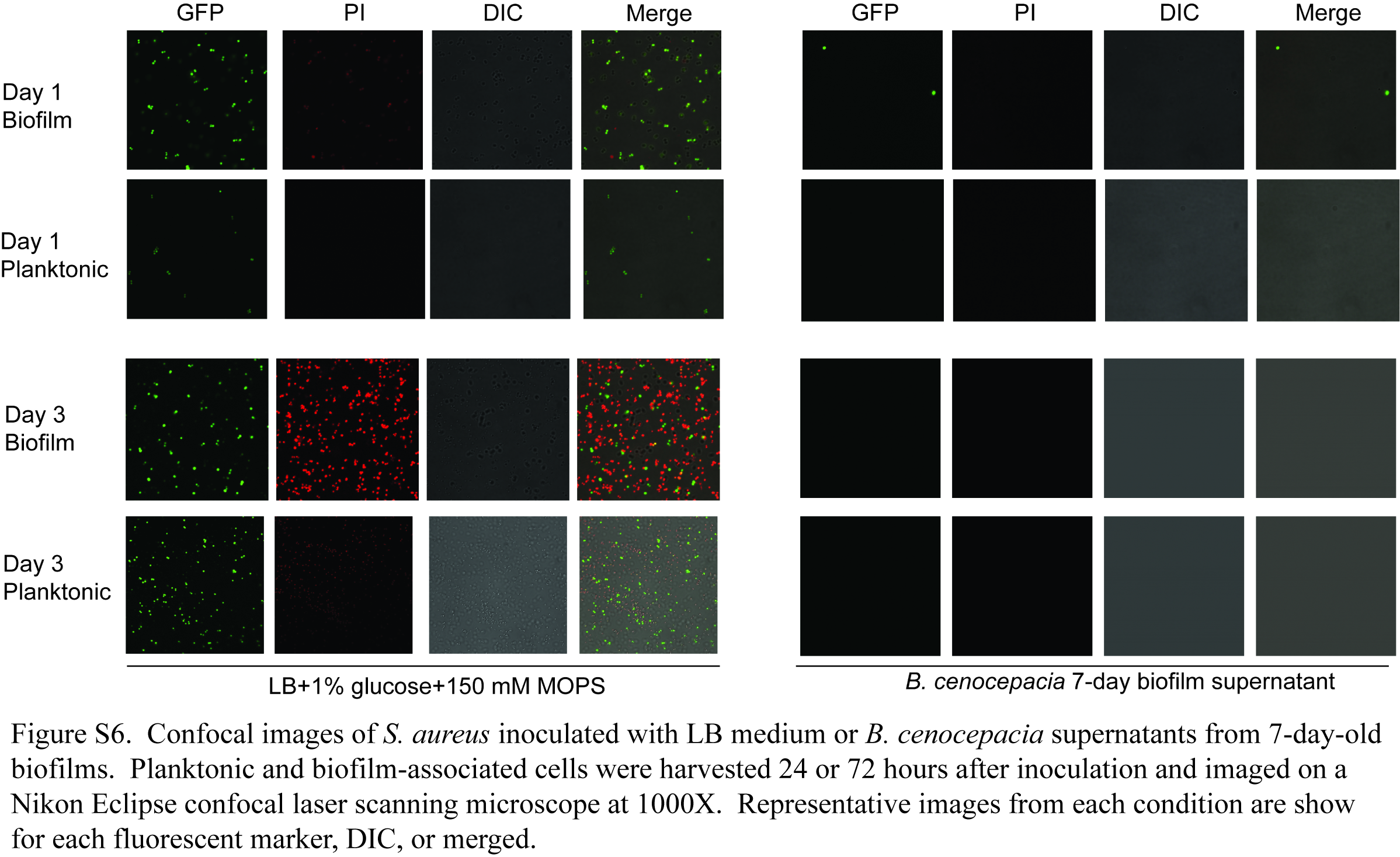

Supplement: Figure S6 — Confocal images. [file jb.00116-23-s0006.tif]
